# Geographical Disparities in Faecal Immunochemical Test‐Based Colorectal Cancer Screening Participation and Positivity Rates: A Systematic Review and Meta‐Analysis

**DOI:** 10.1002/hpja.70168

**Published:** 2026-03-01

**Authors:** Melkalem Mamuye Azanaw, Erin L. Symonds, Geraldine Laven‐Law, Wudneh Simegn Belay, Syme Aftab, Muktar B. Ahmed, Molla M. Wassie

**Affiliations:** ^1^ Department of Medicine, College of Medicine and Public Health Flinders University Bedford Park South Australia Australia; ^2^ Department of Public Health College of Health Sciences, Debre Tabor University Debre Tabor Ethiopia; ^3^ Gastroenterology Department Flinders Medical Centre, Southern Adelaide Local Health Network Bedford Park South Australia Australia; ^4^ South Australian immunoGENomics Cancer Institute, the University of Adelaide Adelaide South Australia Australia; ^5^ Department of Social and Administrative Pharmacy, School of Pharmacy College of Medicine and Health Sciences, University of Gondar Gondar Ethiopia

**Keywords:** colorectal neoplasms, community participation, faecal occult blood test, health status disparities, mass screening

## Abstract

**Background:**

People in rural and remote areas often participate less actively in colorectal cancer (CRC) prevention practices, including faecal immunochemical testing (FIT). However, the evidence on this is limited.

**Objective:**

The aim of this systematic review and meta‐analysis was to summarise geographical disparities in participation and positivity of FIT‐based CRC screening.

**Methods:**

Six databases were searched for articles published until June 2024. We included studies reporting FIT‐based CRC screening among average‐risk individuals aged 40–74, examining geographical disparities using location‐based or geospatial methods. Two reviewers independently screened, assessed bias, and extracted data. Random‐effects models estimated pooled participation, positivity rates, and odds ratios for geographical effects.

**Results:**

Of 8532 articles, 35 were included in the review, with 21 used for meta‐analysis. The overall FIT participation rate was 49.9% (95% confidence interval [CI]: 40.6, 59.2). In Europe, individuals in rural areas had higher participation rates compared to urban areas (pooled odds ratio [POR]: 1.20; 95% CI: 1.01, 1.42), while in Australia, remote areas exhibited lower odds of participation than metropolitan areas (POR: 0.75; 95% CI: 0.65, 0.87). The overall FIT positivity rate was 8.70% (95% CI: 6.50, 11.70), with no significant difference between rural and urban areas (*p* = 0.24).

**Conclusions:**

A notable disparity in the FIT‐based CRC screening participation rate was observed using the geographical definition of rurality and remoteness. Further research is needed to identify the sociocultural, healthcare access, and policy factors driving these differences and develop targeted strategies to improve screening and address barriers for underserved populations.

## 1 Introduction

Globally, colorectal cancer (CRC) is the second leading cause of cancer deaths and the third most diagnosed cancer, with more than 1.9 million new cases and 904 000 deaths in 2022 [1]. The rate of CRC varies by region, with the highest burden in North America, Australia/New Zealand, and Europe [1].

Organised CRC screening has been proven to decrease CRC incidence and mortality [2, 3]. An organised CRC screening program refers to a centrally coordinated approach with a systematic invitation and follow‐up for screening, aimed at boosting participation, ensuring quality, and reducing disparities in CRC outcomes. In contrast, opportunistic screening relies on individuals or providers to initiate testing, often due to inconsistent practice and missed opportunities for screening [4]. As a result, many countries have implemented this organised screening program, significantly decreasing CRC‐related deaths and cases [5, 6]. Screening methods include invasive procedures, such as colonoscopy and flexible sigmoidoscopy, typically performed in clinic settings and non‐invasive home‐based methods, such as faecal occult blood testing (FOBT) and faecal immunochemical testing (FIT) [7, 8]. Of these, FIT is the most widely used and recommended due to its higher specificity, sensitivity, cost‐effectiveness, and accessibility [8, 9, 10, 11]. Despite this, participation rates are often suboptimal, influenced by demographic, behavioural, and environmental factors [12, 13, 14]. Furthermore, COVID‐19 significantly disrupted routine CRC screening services and shifted healthcare resources towards pandemic response. At the start of the pandemic, participation dropped by up to 90%, with colonoscopy‐based screening more affected than FIT‐based screening, as FIT is a home‐based test. These disruptions may have delayed early detection [15, 16].

Global evidence showed disparities in CRC screening between rural and urban populations [17]. Individuals in rural, remote, or deprived areas have exhibited less engagement in CRC prevention practices, including lower FIT participation and longer delays to diagnostic colonoscopy after a positive FIT result [18, 19, 20, 21, 22, 23]. While previous reviews have reported lower screening participation rates in rural areas compared to urban ones [17, 19], they have not examined separate FIT‐based CRC screening, despite FIT being the most recommended screening method. The positivity rate of FIT‐based CRC screening is determined by faecal haemoglobin concentration, with countries using different threshold levels and FIT brands [24, 25]. However, no evidence has been summarised on FIT positivity rates by geography and threshold level. Identifying geographical differences in positivity rates could help guide targeted intervention in areas with higher rates, potentially reflecting a higher burden of unaddressed CRC cases or precancerous conditions.

Although geography has been linked to CRC screening disparities, more focused evidence is needed on FIT‐based participation and positivity rates. One main challenge is the lack of standardised definitions of ‘rurality’, which varies not only across countries but sometimes within countries [26]. In this review, geographical definitions are based on rural–urban distinctions and the Accessibility/Remoteness Index of Australia (ARIA+). Due to the variability in how geography is defined across studies and regions, we grouped the pooled estimates by broad global regions—Europe, the Americas, Asia and Australia—to facilitate a meaningful synthesis. Therefore, this systematic review and meta‐analysis aim to summarise geographical disparities in participation and positivity rates of FIT‐based CRC screening across world regions.

## 2 Methods

### 2.1 Protocol Registration

The review was conducted following the Preferred Reporting Items for Systematic Reviews and Meta‐Analyses (PRISMA) guidelines [27]. The protocol has been registered with the International Prospective Register of Systematic Reviews (PROSPERO) with a registration ID of CRD42024556547.

### 2.2 Search Strategy

Six databases (Medline, CINAHL (EBSCOhost), Scopus (Elsevier), Web of Science, Cochrane, and ProQuest) were searched for primary articles published until June 2024. The search combined subject headings, key terms, and phrases relating to geographic factors and FIT‐based CRC screening. The Table S1 provides full details of the searches.

### 2.3 Definition of Geographical Measures

This systematic review employed various definitions to delineate geographical areas, including population density, the remoteness index, and the rural–urban continuum classification.

For the Australian‐based studies, the remoteness index was used to classify areas into five classes based on the Accessibility/Remoteness Index of Australia (ARIA+), which measures relative geographic access to services. ARIA+ is a continuous scale ranging from 0 (high accessibility) to 15 (high remoteness). The five remoteness classes consist of major cities (0–0.2), inner regional (0.2–2.4), outer regional (2.4–5.92), remote (5.92–10.53), and very remote (> 10.53) [28]. For this review, ‘remote areas’ and ‘very remote areas’ were categorised as remote, while ‘outer regional,’ ‘remote,’ and ‘very remote’ areas were classified as rural.

The rural–urban classification system is used for other regions (Europe, the United States and Asia) [29, 30, 31, 32]. Therefore, studies in these regions were considered based on this definition. Urban areas are typically described as ‘urban’, ‘dense areas’, ‘metropolitan’, and ‘major cities’, whereas rural areas are described as ‘rural’, ‘non‐metropolitan’, or ‘villages’.

### 2.4 Inclusion and Exclusion Criteria

The population, exposure, and outcome framework was applied for this review [33].

Inclusion criteria were:

- Articles that reported separate FIT CRC screening participation and positivity rates among the average‐risk screening population aged 40–74. Separate FIT CRC screening refers to articles that report FIT‐based CRC screening independently, without combining them with other screening modalities (e.g., colonoscopy, sigmoidoscopy, guaiac FOBT)
- Articles that reported results of geographical disparity (remoteness or rurality or least dense, urbanity, metropolitan, or highly dense area/major cities) or analysis using geospatial approaches (geographical regression, geospatial analysis, Bayesian spatial analysis, spatiotemporal analysis, or other descriptive spatial analysis).
- Articles published any time up to June 2024.

Articles were excluded under the following conditions:

- Articles focused on individuals with gastrointestinal symptoms or those at elevated risk for CRC (i.e., due to inflammatory bowel disease, Lynch Syndrome, hereditary conditions, or prior colorectal neoplasia).
- Articles conducted in a single geographical area without comparisons (such as only in urban areas or only in rural areas).
- Articles using non‐FIT screening methods (e.g., guaiac FOBT, colonoscopy) or unclear screening methods.
- Reviews, qualitative articles, opinion papers, articles without full‐text access, and non‐English publications.

### 2.5 Study Outcomes

The primary outcomes of this review were the pooled participation and positivity rates in FIT‐based CRC screening by geography. The FIT participation rate was defined as the proportion of eligible individuals who returned the FIT sample divided by the number of individuals invited at the specified time. The FIT positivity rate was the proportion of individuals with FIT results at or above the determined haemoglobin positivity cut‐off level, divided by the number of individuals with an assessable stool sample, which might be one sample or two samples [8]. The positivity thresholds vary substantially across countries, ranging from 8.5 to 120 μg haemoglobin/g faeces, with 20 μg/g being the commonly used cut‐off [24].

### 2.6 Selection Process

All articles identified through electronic database searches were first imported into the EndNote software (Clarivate Analytics, 2013) to remove duplicates. The searches were then imported into Covidence (Veritas Health Innovation, Melbourne, Australia) for further duplicate removal, title and abstract screening and full text review. Study quality was assessed using a modified Newcastle‐Ottawa Scale (NOS) tailored for cohort and cross‐sectional study designs. The assessment focused on three main domains: selection, comparability, and outcomes. Each domain includes specific criteria and studies are awarded points based on how well they meet these standards. Overall scores were classified as follows: 9–10 points (excellent), 7–8 points (good), 5–6 points (satisfactory) and 0–4 points (unsatisfactory) [34, 35]. Full details of the assessment criteria are provided in the Table S3. Two reviewers independently screened and assessed the quality of articles, with discrepancies resolved by a third reviewer.

### 2.7 Data Collection Process

Data were systematically extracted from all included studies using a structured framework. Extracted data included general study details (e.g., study ID, author's last name and year of publication), population characteristics (e.g., age), study methods (e.g., study design, data sources), screening outcomes (e.g., invited, screened, positive tests by geography) and geospatial analysis features (e.g., spatial autocorrelation). For studies reporting multiple rounds of FIT screening, data were extracted and reported separately for each round (e.g., Round 1 [R1], Round 2 [R2], Round 3 [R3]), based on the respective data collection periods. When the types of screening methods used were unclear or unreported, we attempted to contact the corresponding authors via email to obtain clarification. Authors were contacted up to two times over an 8‐week period, and responses were received from six studies. When no response was obtained, we excluded the articles (Table S2). Two reviewers independently conducted data extraction, and any disagreements were resolved through review team discussion until a consensus was achieved.

### 2.8 Data Synthesis

Data were analysed using R (version 4.4.1) software, with the ‘meta’ and ‘metafor’ packages to calculate the pooled participation rates, positivity rates, and odds ratios. A systematic review, subgroup meta‐analysis, and meta‐regression were conducted. Subgroup meta‐analyses by region, data collection period, stool samples, test delivery methods, and study quality explored geographical disparity. These characteristics were selected based on their relevance to the research question and the availability of data across included studies. Given the variability in data reporting on these characteristics, subgroup analyses were applied flexibly to ensure accurate comparisons. This analysis aimed to reduce heterogeneity and enhance the interpretability of findings within each group. Heterogeneity was assessed using I^2^ [36] and forest plots displayed study results with 95% confidence intervals (CI). Funnel plots and Egger's test assessed publication bias [37, 38]. A sensitivity analysis was conducted to check the presence of outliers. Meta‐regression was performed using a random‐effects model to examine the source of heterogeneity across studies. Study‐level characteristics, such as data sources, year of publication, and study quality, were used to determine whether they are associated with variations in effect size reported across studies [39]. For the meta‐analysis, we used raw data from studies that provided complete geographical and screening details (e.g., rural–urban classification and participation counts). If a study reported on multiple rounds of FIT, then the results were reported and included for each round separately. A pooled odds ratio (POR) with a 95% confidence interval (CI) was used to assess the effects of geography on participation. A statistically significant result was defined as a *p* < 0.05.

## 3 Results

### 3.1 Search Results

**FIGURE 1:**
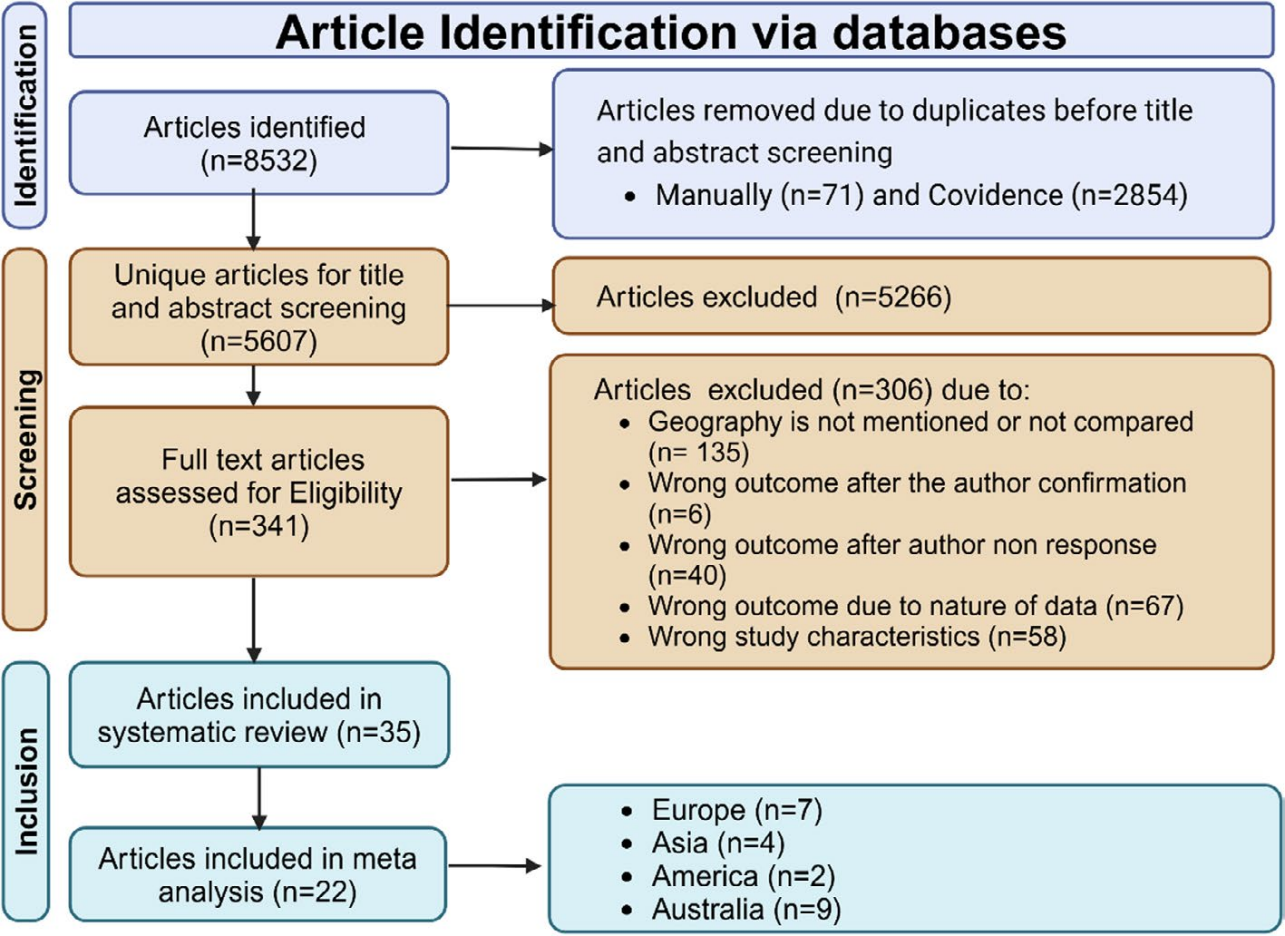
Flow chart of selection of articles for the systematic review using PRISMA checklists.

The search identified 8532 articles from six databases. After removing 2925 duplicates and excluding 5607 based on title and abstract screening, 341 articles were available for full‐text screening. Of these, 306 were excluded for various reasons (Table S2), leaving 36 articles that met the inclusion criteria [18, 22, 40, 41, 42, 43, 44, 45, 46, 47, 48, 49, 50, 51, 52, 53, 54, 55, 56, 57, 58, 59, 60, 61, 62, 63, 64, 65, 66, 67, 68, 69, 70, 71, 72, 73]. One study [72] was later excluded due to an overlapping study population with another study [71], which was retained for its larger sample size. Four articles reported separate FIT participation rounds, all of which were included in the analysis [22, 50, 59, 66] (Figure 1).

### 3.2 Descriptive Characteristics of the Included Articles

#### 3.2.1 General Characteristics of the Included Articles

The 35 articles were found across four world regions: Europe, Australia, Asia, and America and represented 16 countries: the Czech Republic, Wales, France, Australia, the Netherlands, Italy, Japan, China, the USA, Romania, Iran, Spain, Malaysia, South Korea, Bulgaria, and Belgium. Most articles were from Europe (*n* = 14) [18, 41, 42, 45, 49, 51, 54, 58, 59, 64, 67, 68, 69, 73] and Australia (*n* = 11) [22, 40, 43, 47, 48, 50, 55, 63, 65, 70, 71], followed by Asia (*n* = 7) [44, 46, 52, 56, 61, 62, 66], and the Americas (*n* = 3) [53, 57, 60]. Nearly two‐thirds (65.7%) were from countries with organised CRC screening programs [18, 22, 40, 41, 42, 43, 45, 46, 47, 48, 50, 51, 52, 54, 55, 56, 58, 63, 64, 65, 66, 67, 71]. Over half of the articles used postal mail to return FIT samples [18, 22, 40, 42, 43, 45, 49, 50, 51, 52, 55, 56, 57, 58, 60, 63, 64, 65, 68, 71]. Sixteen articles reported the number of sample tests: eight used one sample FIT [41, 42, 49, 60, 61, 62, 67] and eight used a two‐sample FIT [18, 22, 40, 46, 47, 48, 50, 56, 65]. Regarding study design, over two‐thirds (77.7%) were cross‐sectional/survey [18, 22, 40, 41, 43, 44, 45, 46, 47, 48, 50, 52, 53, 55, 56, 59, 61, 63, 64, 65, 66, 67, 68, 69, 70, 71, 73] (Table 1).

**TABLE 1:** Summary of included articles for geographical disparities in faecal immunochemical test‐based colorectal cancer screening participation and positivity Rates (*n* = 35).

Characteristics of including articles			Frequency	Percentage
General information				
Regional classification		Europe	14	40.0
		Australia	11	31.4
		Asia	7	20.0
		America	3	8.6
COVID‐19 period		Yes	7	20.0
		No	28	80.0
Population characteristics				
Target age limit in years (50–74 years)		Yes	18	51.4
		No^a^	18	51.4
Methodology				
		Cross‐sectional/survey	27	77.7
		Follow‐up/cohort	8	22.3
Data sources		Administrative collections^b^	21	60.0
		Research survey	14	40.0
Methods of analysis	Spatial	Mapping/Hotspot/cluster	3	8.6
		Multiscale GWR	1	2.9
		Multilevel analysis	3	8.6
		Bayesian spatial models	1	2.9
	Non‐spatial	Descriptive	11	31.4
		GEE	1	2.9
		Logistic	13	37.1
		Linear	1	2.9
		Ordinal	1	2.9
FIT sample numbers^c^		One	8	22.8
		Two	8	22.8
		Not stated	19	54.4
FIT positivity threshold (μg Hb/g faeces)		≥ 20	12	34.3
		< 20	7	20.0
		Not stated	16	45.7
Organised population‐based		Yes	23	65.7
		No	12	34.3
Test delivery method		Postal mail	20	57.1
		Self‐reported	5	14.3
		Other (family physician, research team)	7	20.0
		Not stated	3	8.6
Geographical classification measures		ARIA+ classification	11	31.4
		Population density	14	40.0
		RUC	10	28.6

Abbreviations: ARIA+, accessibility/remoteness index of Australia; GEE, generalised estimating equations; GWR, geographically weighted regression; RUC, rural–urban continuum.

^a^ Articles used different ages of 40–64, 45–75, 50–70, ≥ 50, ≥ 45, 61–75, 55–65, specific age.

^b^ Administrative collections include population‐based surveys, national cancer registries, health insurance data, census, and clinical databases.

^c^ FIT number sample is the number of faecal samples collected, with one sample with single stool sample and two samples two separate samples collected on different days.

#### 3.2.2 Study Quality Assessment

All studies were rated with modifiable NOS, scoring between five and ten points. Most articles (88.6%) were rated as excellent [22, 41, 42, 43, 48, 56, 58, 59, 62, 63, 65, 71] and good [18, 40, 44, 46, 47, 49, 50, 51, 52, 54, 55, 57, 60, 61, 67, 69, 70, 73]. Four articles were rated as satisfactory [53, 64, 66, 68], and none were assessed as unsatisfactory (Table S3).

#### 3.2.3 Geographical Disparity in FIT‐Based Participation Rate

Of the 35 articles, 28 reported participation rates, with 33 specific data collection periods available for analysis. Out of the 15 articles that reported adjusted odds ratio/relative risk for the FIT participation [18, 41, 43, 44, 45, 47, 48, 49, 51, 55, 58, 59, 62, 69, 71]: seven reported higher odds of participation in less dense or rural areas [49, 54, 58, 59, 67, 68, 69], while two found higher odds in high‐density or urban areas [43, 51]. Six articles found no significant difference between geographical areas [41, 47, 48, 55, 62, 71]. Additionally, two articles reported relative risk estimates, indicating higher participation rates in less densely populated areas compared to high‐density areas [18, 45] (Table 2, Figures S1 and S2).

**TABLE 2:** Characteristics of included articles for faecal immunochemical test‐based colorectal cancer screening participation rates.

Geographical disparity of participation rates by population density, rural–urban, and remoteness index										
Study references	Country	Age range, years	Data sources	Data collection period	Number of FIT samples	Geographical measure definitions	Comparison of geographical area	Adjusted variables		Highest participation rate
								Additional covariates	OR (95% CI)	
Bright et al. [41]	Wales	60–74	Administrative	2020–2021	Two	Rural–urban	**Rural** vs. Urban	Ethnic group, age, sex, income deprivation	Adjusted: 0.99 (0.96, 1.04)	No difference
de Klerk et al. [18]	Netherlands	55–75	Administrative	2014–2016	One	Population density	**Less dense** vs. High dense	N/A	Adjusted RR: 1.23 (1.23–1.24)	Less dense
Giorgi Rossi et al. [45]	Italy	50–69	Administrative	2005–2014	N/A	Population density	**Metropolitan** vs. non‐metropolitan	Geographical area, year, screening round	Adj: RR: 0.35 (0.27, 0.45)	Less dense
Hol et al. [49]	Netherlands	50–74	Research survey	2006–2007	One	Rural–urban	**Rural** vs. Urban	Sex, age, and SES	Adj: 2.3 (1.6 to 3.3)	Rural
Kregting et al. [51]	Netherlands	55	Administrative	2014–2019	N/A	Rural–urban	**Rural** vs. Urban	SES/household income timing of invitations	Adj: 0.75 (0.74, 0.77)	Urban
		60	Administrative	2014–2019	N/A	Rural–urban	**Rural** vs. Urban	SES/household income timing of invitations	Adj: 0.77 (0.76, 0.78)	Urban
Manuc et al. [54]	Romania	50–74	Administrative	2022–2023	N/A	Rural–urban	**Rural** vs. Urban	N/A	Cru: 1.47 (1.43, 1.52)	Rural
Pornet et al. [58]	France	50–74	Research survey	2007–2010	N/A	Rural–urban	**Rural** vs. Urban	Sex, age, health insurance plan, source of invitation	Adj: 1.06 (1.00–1.34)	Rural
Cebrino [59]	Spain	50–69	Administrative	2017–2020	N/A	Rural–urban	**Rural** vs. Urban	Age, level of education, social class	Adj: 1.14 (1.07–1.22)	Rural
Tsvetanova Dimova et al. [67]	Bulgaria	45+	Research survey	2013	One	Rural–urban	**Rural** vs. Urban	N/A	Cru: 2.17 (1.42, 3.31)	Rural
Roosbroeck et al. [69]	Belgium	50–74	Research Survey	2009	N/A	Rural–urban	**Rural** vs. Urban	Invitation strategy and gender	Adj: 2.90 (2.66, 3.16)	Rural
Van Hal et al. [68]	Belgium	50–74	Research Survey	2009	N/A	Rural–urban	**Rural** vs. Urban	N/A	60.9% in rural and 34.3% in urban	Rural
Dasgupta et al. [22]	Australia	50–74	Administrative	2015–2016	Two	ARIA+	**Remote** vs. Metropolitan	NA	Crude: 0.81 (0.80, 0.83)	Metropolitan
Dasgupta et al. [22]	Australia	50–74	Administrative	2017–2018	Two	ARIA+	**Remote** vs. Metropolitan	NA	Crude: 0.73 (0.72, 0.74)	Metropolitan
Dasgupta et al. [22]	Australia	50–74	Administrative	2019–2020	Two	ARIA+	**Remote** vs. Metropolitan	NA	Crude: 0.66 (0.65, 0.67)	Metropolitan
Fletcher [43]	Australia	50–74	Administrative	2020	Two	ARIA+	**Rural** vs. Metropolitan	Age, gender, and SES	Adj: 0.92 (0.84, 0.99)	Metropolitan
Goodwin et al. [47]	Australia	50–74	Research survey	2017–2018	Two	ARIA+	**Outer and remote** vs. Metropolitan	Age	Adj: 1.23 (0.68, 2.23)	No difference
Goodwin et al. [48]	Australia	50–74	Administrative	2014–2015	Two	ARIA+	**Rural** vs. Metropolitan	Age	Adj: 1.06 (0.87–1.30)	No difference
Irwin et al. [50]	Australia	50–74	Administrative	2019	Two	ARIA+	**Remote** vs. Metropolitan	N/A	Cru: 0.73 (0.67, 0.79)	Metropolitan
Irwin et al. [50]	Australia	50–74	Administrative	2020	Two	ARIA+	**Remote** vs. Metropolitan	N/A	Cru: 0.73 (0.66, 0.80)	Metropolitan
Martini et al. [55]	Australia	55–65	Administrative	2007–2008	N/A	ARIA+	**Remote** vs. Metropolitan	Sex, age	Adj: 1.03 (0.94–1.12)	No difference
Sun et al. [65]	Australia	50–74	Administrative	2011–2013	Two	Remoteness Area by LGAs	**Remote** vs. Metropolitan	N/A	Cru: 0.74 (0.72, 0.77)	Metropolitan
Varlow et al. [70]	Australia	50+	Administrative	2011	N/A	Geographic region	**Rural** vs. Metropolitan	N/A	Cru: 1.22 (0.99, 1.49)	No difference
Ward [71]	Australia	55, 65	Administrative	2007–2008	Two	ARIA+	**Remote** vs. Metropolitan	Sex and ISRD	Adj: 1.03 (0.94–1.12)	No difference
Schliemann et al. [62]	Malaysia	50–75	Administrative	2018	One	Study sub‐district	**Rural** vs. urban	Age, gender, ethnicity, education, working status, monthly household income, cancer history	Adj: 1.09 (0.75, 1.59)	No difference
Fukuda et al. [44]	Japan	40–64	Research survey	2001	N/A	Population density	**Rural** vs. urban	Age, marital status, occupation, income	Adj: 0.69 (0.53, 0.90)	Rural
Lin et al. [52]	China	50–74	Administrative	2015–2017	Two	N/A	**Rural** vs. urban	N/A	Cru: 1.07 (1.06, 1.08)	Rural
Trinh et al. [66]	Korea	50+	Administrative	2019	N/A	Geographic regions	**Rural** vs. urban	N/A	Cru: 0.73 (0.55, 0.97)	Urban
Trinh et al. [66]	South Korea	50+	Administrative	2020	N/A	Geographic regions	**Rural** vs. urban	N/A	Cru: 0.52 (0.37, 0.74)	Urban
LoConte et al. [53]	USA	50–79	Research survey	2010–2012	N/A	RUC	**Rural** vs. urban	N/A	Cru: 1.04 (0.58, 1.86)	No difference
O'Connor et al. [57]	USA	50–74	Research survey	2014–2015	N/A	Population density	**Rural** vs. urban	N/A	Cru: 0.77 (0.72, 0.83)	Urban

Geographical disparities of participation rates at the local area levels								
						Area level	Spatial analysis	Geographical level factors
Baum [40]	Australia	50–74	Administrative	2018–2020	Two	SA3	Multiscale Geographically Weighted Regression	Indigenous population (Mean ± SD: −0.56 ± 0.25) Poor English skills (Mean ± SD: −0.24 ± 0.09) Volunteer individuals (Mean ± SD: 0.31 ± 0.08) and Not engaged in education or employment (Mean ± SD: 0.46 ± 0.07)
Dasgupta et al. [22]	Australia	50–74	Administrative	2015–2020	Two	SA2	Bayesian model	Lower participation for remote areas compared to major cities and regional areas
de Klerk et al. [18]	Netherlands	55–75	Administrative	2014–2016	One	Postcodes	Mapping	Shows the difference in participation rates by urban density
Martini et al. [55]	Australia	55 &65	Administrative	2007–2008	N/A	Postcodes	Ecological analysis	The pattern of participation varies in rural and remote areas
Ramai et al. [60]	USA	> = 50	Administrative	2014–2016	N/A	Census tract level	Hot spot/cluster analysis	Hot‐spot analysis indicates the presence of statistically significant spatial clusters of low screening levels in individuals below the poverty level and individual data points that concentrate on areas of greater poverty.
Slimings and Moore [63]	Australia	50–74	Administrative	2016–2017	Two	LGA	Multilevel analysis	Participation ranged from 21.4% to 48.0% by LGAs Remote or very remote LGAs dominated the lowest rates.
Ward [71]	Australia	55 &65	Research survey	2007–2008	N/A	Postcodes	Mapping	For both genders, participation varied by postcode, There was a disparity in participation in rural and remote areas.
Stracci et al. [64]	Italy	50–74	Research survey	2006–2012	N/A	Census tract	Multilevel analysis	Family doctor practices and the percentage of foreign‐born people in practice were significant clustering factors.

*Note:* Written in bold are exposed groups.

Abbreviations: adj, adjusted; ARIA+, accessibility/remoteness index of australia (metropolitan, inner regional, outer regional, remote, and very remote), high dense indicates ≥ 1000 persons/km^2^ and less dense indicates ≤ 500 individuals/km^2^; ASGS, Australian statistical geography standard; cru, crude; IRSD, index of relative social disadvantage; LGA, local government areas; LGA, local government areas; N/A, not applicable; N/A, not applicable; OR, odds ratio; RR, relative risk; SA 2/3, Statistical area 2/3 areas; SD, standard deviation; SES, socioeconomic status.

Eight articles used geospatial analysis at the local area level, applying geographical units, such as the statistical areas [22, 40], postcodes [55, 71], local government areas [63], and census tracts [60, 64]. Participation patterns varied, with the identification of statistically significant spatial clusters [22, 60, 63] (Table 2).

#### 3.2.4 Geographical Disparity in FIT Positivity Rate

Nine articles (13 data collection periods) reported FIT positivity rates [42, 46, 52, 53, 54, 56, 61, 69, 73]. Three articles indicated higher positivity rates in less densely populated or rural areas compared to high‐density or urban areas [46, 53, 73]. In contrast, two articles showed higher positivity rates in highly dense or urban areas [52, 73], and four articles found no statistically significant difference [42, 54, 56, 73]. Notably, only seven articles provided raw data for comparing rural and urban areas [42, 46, 52, 53, 54, 56, 73] (Table 3).

**TABLE 3:** Characteristics of included articles for faecal immunochemical test‐based colorectal cancer screening positivity rates.

Study references	Country	Age range, years	Data sources	FIT brand	FIT threshold	Geographical measure definitions	Geographical classification	Adjusted variables		Highest positivity rate
								Additional covariates	OR (95% CI)	
Belobradek et al. [73]	Czech Republic	50–69	Administrative	Sentinel diagnostics	15 μg Hb/g	Rural–urban	Rural vs. Urban	N/A	Cru: 0.96 (0.93, 0.99)	Urban
Belobradek et al. [73]	Czech Republic	50–69	Administrative	Sentinel diagnostics	15 μg Hb/g	Rural–urban	Rural vs. Urban	N/A	Cru: 1.01 (0.98, 1.04)	No difference
Belobradek et al. [73]	Czech Republic	50–69	Administrative	Sentinel Diagnostics	15 μg Hb/g	Rural–urban	Rural vs. Urban	N/A	Cru: 1.08 (1.05, 1.10)	Rural
Dancourt et al. [42]	France	50–74	Research survey	OC‐Sensor	100 ng/mL	N/A	Rural vs. Urban	Sex, age, season, sample return time	1.1 (0.9, 1.3)	No difference
Gong et al. [46]	China	50–74	Administrative	WPHM hemosure	100 ng/mL	n/a	Rural vs. Urban	Age, marital status, education, occupation, and screening method	1.4 (1.39–1.47)	Rural
Lin et al. [52]	China	50–74	Research survey	WPHM hemosure	20 μg Hb/g	N/A	Rural vs. Urban	n/a	Cru: 0.78 (0.76, 0.81)	Urban
LoConte et al. [53]	USA	50–79	Research survey	OC‐sensor	20 μg Hb/g	RUC	Rural vs. Urban	N/A	Cru: 2.39 (1.25, 4.57)	Rural
Manuc et al. [54]	Romania	50–74	Administrative	OC‐sensor	20 μg Hb/g	RUC	Rural vs. Urban	N/A	Cru: 0.92 (0.88, 0.96)	No difference
Nikbakht et al. [56]	Iran	50+	Research survey	Haemoglobin ELISA Kit	20 μg Hb/g	RUC	Rural vs. Urban	Sex, age group, education, marital status, and BMI	0.85 (0.61–1.18)	No difference
Salimzadeh et al. [61]	Iran	45–75	Administrative	Haemoglobin ELISA Kit	20 μg Hb/g	N/A	Rural vs. Urban	N/A	N/A	N/A
Tsvetanova Dimova et al. [67]	Belgium	50–74	Research survey	OC‐sensor	75 ng/mL	N/A	N/A	N/A	N/A	N/A

Abbreviations: μg, micro gram; adj, adjusted; BMI, body mass index; cru, crude; Hb, haemoglobin; N/A, not applicable; N/A, not applicable; OR, odds ratio.

### 3.3 Meta‐Analysis of Included Articles

#### 3.3.1 FIT‐Based Participation Rates

##### 3.3.1.1 Overall FIT‐Based Participation Rates by Geographical Regions

Across articles from 16 countries, 22 000 413 individuals were invited for screening, and 9 397 611 participated. The overall FIT‐based participation rate was 49.9% (95% CI: 40.6–59.2; *I* ^2^ = 100%), ranging from 15.1% [57] to 96% [61]. Participation rates also varied by region: 45.2% in Australia (*I* ^2^ = 100%), 47.0% in America (*I* ^2^ = 99.9%), 36.4% in Asia (*I* ^2^ = 99.7%), and 62% in Europe (*I* ^2^ = 100%) (Table S4).

##### 3.3.1.2 Effects of Geographical Disparity on FIT‐Based Participation Rates

Twenty‐one articles provided complete data to calculate the odds ratio for FIT‐based participation. Twelve reported rural–urban classifications [18, 41, 44, 51, 52, 53, 54, 57, 58, 59, 62, 66, 67], and nine remoteness area classifications [22, 43, 47, 48, 50, 55, 65, 70]. Due to regional differences in geographical definitions, separate meta‐analyses were conducted to minimise heterogeneity.

**FIGURE 2:**
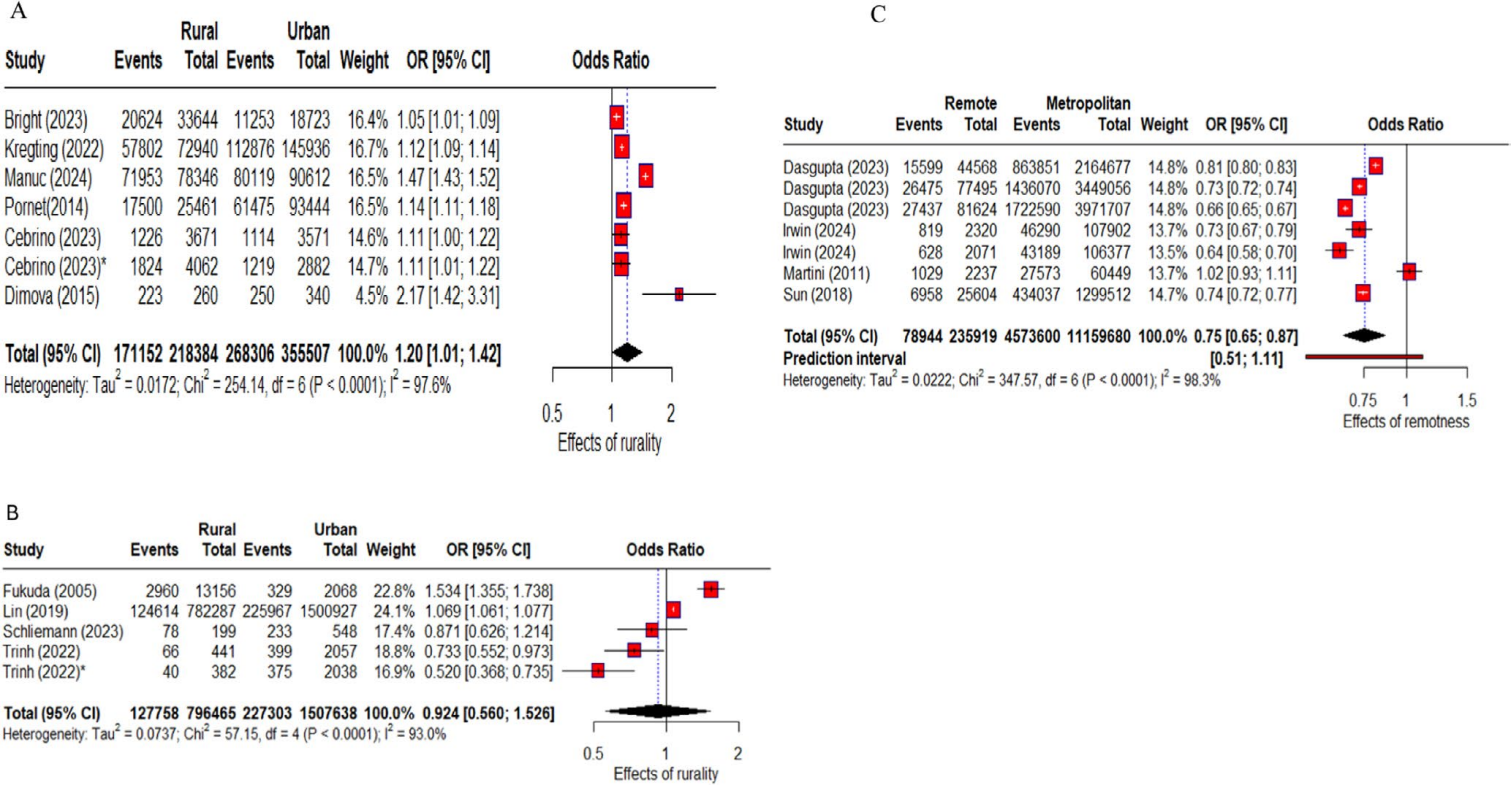
Forest plot of random effects meta‐analysis of effects of population density (A), rurality (B), and remoteness (C) on FIT‐based colorectal cancer screening participation rate in Europe, Asia, and Australia, respectively.

In *Europe*, individuals in rural areas had 20% higher participation rates compared to those in urban areas (pooled odds ratio [POR]: 1.20; 95% CI: 01, 1.42; *I* ^2^ = 97.60%), with no evidence of publication bias (*p* = 0.82) and outliers (Figures 2A and S3, Table S5).

Only two articles provided complete rural–urban data in the *United States*, and their sample sizes differed significantly (633 vs. 30 667), making it challenging to conduct a meta‐analysis (Table S5).

In *Asia*, no significant difference was found between rural and urban areas (POR: 0.92; 95% CI: 0.56, 1.53; *I* ^2^ = 93.0%), with no evidence of publication bias (*p* = 0.79) (Figures 2B and S4, Table S5).

In *Australia*, nine articles (11 data collection periods) provided raw data for calculating the odds ratio based on the remoteness index. The participation rate was 34.3% in remote areas and 44.9% in metropolitan areas (Table S6). Individuals in remote areas had 25% lower odds of participating than those in metropolitan areas (POR: 0.75; 95% CI: 0.65, 0.87; *I* ^2^ = 98.3%) (Figure 2C), with no evidence of publication bias (*p* = 0.67). In contrast, participation in inner regional areas was 14% higher than in metropolitan areas (POR: 1.14; 95% CI: 1.08, 1.19; *I* ^2^ = 97.4%), with no evidence of publication bias (*p* = 0.62) (Figure S5).

##### 3.3.1.3 Summary of Subgroup Analysis Using Different Characteristics

In *Europe*, the participation rate was significantly higher in rural areas compared to urban areas in excellent‐quality articles (POR: 1.10; 95% CI: 1.04, 1.16; *I* ^2^ = 75.2%), but not in good‐quality articles (POR: 1.42; 95% CI: 0.68, 2.93; *I* ^2^ = 99.80%). No significant group difference was observed in this quality classification (*p* = 0.14) (Table S7).

In *Asia*, rural areas had a significantly lower participation rate than urban areas between 2015 and 2024 (POR: 0.69; 95% CI: 0.53, 0.92; *I* ^2^ = 56.6%). No statistically significant difference was observed for the 2005–2014 (POR: 1.27; 95% CI: 0.89, 0.89, 1.74; *I* ^2^ = 96.8%). A significant subgroup difference was found between these two time periods (*p* = 0.009) (Table S7).

In *Australia*, subgroup analysis by data collection period revealed a significant difference between 2005–2014 and 2015–2024 for both remote versus metropolitan areas (*p* < 0.001) and inner‐regional regions versus metropolitan areas (*p* = 0.001). There was also a significant subgroup difference between these two time periods (*p* = 0.01).

A meta‐analysis of good‐quality articles (POR: 1.15; 95% CI: 1.13, 1.18; *I* ^2^ = 55.9%) and excellent‐quality articles (POR: 1.13; 95% CI: 1.10, 1.17; *I* ^2^ = 98.1%) revealed significantly higher participation in inner‐regional areas compared to metropolitan areas. Conversely, participation was significantly lower in remote areas than in metropolitan areas, both before COVID‐19 (POR: 0.71; 95% CI: 0.64, 0.98; *I* ^2^ = 98.9%) and during the pandemic (POR: 0.82; 95% CI: 0.64, 0.78; *I* ^2^ = 95.8%). In inner regional versus metropolitan areas, significant subgroup differences were observed based on study setting classification (*p* = 0.01) and data sources (*p* = 0.01) (Table S7).

##### 3.3.1.4 Meta‐Regression of Effects of Rurality and Remoteness

The meta‐regression was conducted in articles from Europe and Australia to explain more of the observed heterogeneity (Table S8).

Differences in data sources and study quality largely explained the heterogeneity in Europe, explaining approximately 49% of the variance in the effects of rurality (*R* ^2^ = 0.49, *τ* ^2^ = 0.04). In multivariable analysis, excellent‐quality articles were associated with higher participation rates (*p* = 0.01) (Table S9).

In Australia, factors such as data sources, the COVID‐19 period, and the test delivery methods explained the variability in the effects of remoteness on FIT‐based participation rates (*R* ^2^ = 0.76, *τ* ^2^ = 0.009) (Table S9).

#### 3.3.2 Meta‐Analysis of FIT Positivity Rate

Twelve articles (14 data collection periods) from 12 countries reported FIT positivity ranging from 4.0% [42] to 25% [56]. The overall positivity rate was 8.7% (95% CI: 6.5%, 11.7%) with varying FIT threshold levels (Figure S7). Positivity rates differ significantly by region (*p* = 0.006), FIT brand (*p* = 0.001) and study quality (*p* = 0.036). Sentinel diagnostics FIT brands showed a higher positivity rate (9.4%) than the OC‐Sensor (7.7%) (Table S10).

**FIGURE 3:**
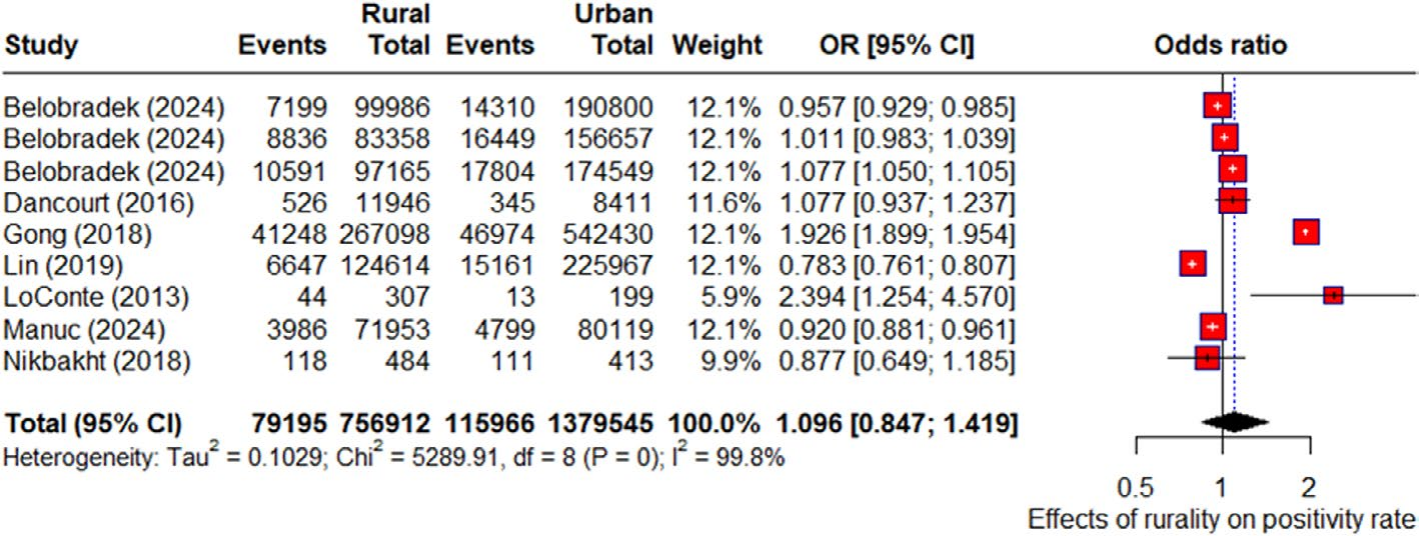
Forest plot of random effects meta‐analysis for effects of rurality on FIT‐based CRC screening positivity rate.

Across seven countries, 221 264 individuals were tested, with positivity rates of 10.1% in rural areas and 8.5% in urban areas. However, a meta‐analysis showed no significant difference (POR: 1.11; 95% CI: 0.83, 1.47; *I* ^2^ = 99.8%) (Figures 3 and S6, Table S11).

## 4 Discussion

To the best of our knowledge, this is the first systematic review and meta‐analysis to summarise geographical disparity in FIT‐based CRC screening participation and positivity rates using international evidence. Previous reviews have focused on articles that assessed CRC screening using mixed tests but did not specifically compare FIT‐based participation and positivity rates by population density, rurality, and remoteness.

This systematic review and meta‐analysis revealed significant geographical disparities in participation in FIT‐based CRC screening in Europe and Australia, but not in other regions. In Australia, participation rates were lower in remote areas compared to metropolitan areas, while in Europe, individuals from rural areas had higher participation rates. FIT positivity rates did not vary by geography, suggesting the test results are not influenced by geographical location. Furthermore, subgroup analyses indicated that studies conducted after 2018 exhibited a significant difference in participation patterns between studies conducted before and after, particularly during the COVID‐19 period, exhibiting greater rural–urban differences, suggesting contextual and temporal influences on screening uptake.

Participation in FIT‐based CRC screening varied by region, ranging from 36.4% in Asia to 61.9% in Europe. This variation may be influenced by the types of screening approach (organised or opportunistic), with organised screening programs standard in Europe and Australia. In contrast, opportunistic screening is more common in the USA and many Asian countries [74]. The opportunistic approach often relies on healthcare providers offering screening tests to individuals during routine visits, with less emphasis on systematically reaching broader populations [75, 76]. In particular, the higher participation rate in Europe may result from the recommendation of FIT as the first‐line test from the European Union guidelines for its member countries [5, 77].

This review explores regional variations in FIT‐based CRC screening participation within the countries to facilitate meaningful comparison. In Europe, participation was higher in rural areas, in contrast to a previous study conducted in the USA, which showed higher urban participation [17]. However, direct comparisons should be made cautiously due to differences in healthcare systems. European countries typically implemented organised population‐based screening programs, while the USA relies more on opportunistic screening. Additionally, the higher cost of healthcare in the USA may limit access and uptake, further influencing participation. Several factors may also explain the geographical disparity of FIT‐based CRC screening in Europe. Evidence showed that individuals from rural areas are targeted for health initiatives, including mobile screening units or outreach programs [78]. European public health authorities are increasingly focused on enhancing outreach programs and campaigns [79]. Residents in these areas may also view other screening methods as more invasive or less accessible, making them more inclined to utilise the non‐invasive and less resource‐demanding FIT test as a practical first‐step screening [80]. In contrast, urban areas offer more private healthcare options, where populations may opt for alternative screening methods perceived as more accurate. Additionally, rural areas bear a higher burden of chronic conditions, including CRC, heightening the perceived need for preventive screening [81, 82]. In urban areas, the diverse population in terms of ethnicity may result in lower participation, with non‐white individuals being less knowledgeable about CRC prevention [5, 76, 83].

This review revealed that overall FIT‐based participation in Asia is significantly lower than in other regions, with no notable difference between rural and urban areas. In contrast to other regions, the ideal screening methods for CRC in Asia remain unclear, and screening uptake is low in many Asia countries due to resource constraints. Government support and healthcare access are limited in rural areas, with only Taiwan and South Korea offering free mass screening, while other countries lack organised population‐based screening programs [84, 85].

The odds of FIT‐based participation in Australia were lower in remote areas but higher in inner‐regional areas compared to metropolitan areas. This finding is consistent with the previous studies [19, 86, 87]. The possible explanation for this geographical disparity is that people in remote areas may have lower awareness, less education, and less exposure to public health messages and media [88]. The lower participation rates in remote areas might be due to the inability to deliver FIT kits by post, since most populations in remote areas lack valid postal addresses [89].

The overall FIT‐based positivity rate was higher, with notable regional variation, ranging from a low positivity rate in the Netherlands to higher rates in Iran. Our finding indicates slightly higher FIT positivity rates compared to those reported in the recent meta‐analysis, which documented an overall positivity rate of 7.28% (95% CI: 6.81%–7.76%), with a broad range from 1.09% to 30.01% and substantial heterogeneity (*I* ^2^ = 99.9) [90]. The heterogeneity in that meta‐analysis was attributed mainly to variations in study number and cutoff thresholds; however, rural–urban differences were not examined as a potential source of variability. In contrast, our review specifically assessed both overall and rural–urban differences and found no significant differences in FIT positivity rates between rural and urban areas. This suggests that the test's performance remains consistent across geographical areas. This implies that observed disparities in screening outcomes are more likely driven by participation‐related and programmatic factors, rather than by intrinsic differences in test performance based on place of residence.

This review also examines the relative impact of small‐area variations in FIT‐based participation in the USA and Australia, utilising advanced geospatial analysis, such as Bayesian spatial models and geographically weighted regression analysis. In the USA, hotspot analysis identified clusters of low screening uptake in small urban areas, while Australian studies identified low participation at the local area level across both rural and remote areas. Evidence showed that targeted health promotion interventions at the small area level proved more effective and feasible than in national or large‐scale geographical areas [91, 92].

This review also examined the sources of heterogeneity through subgroup analysis and meta‐regression across Europe, Asia, and Australia, revealing region‐specific patterns. In Europe, geographical differences in participation were influenced by the study quality classification. In Asia, heterogeneity was primarily driven by the timing of the data collection period, particularly during the COVID‐19 pandemic, and variations in study quality. In Australia, although heterogeneity was present, participation patterns remained consistent across subgroups, regardless of data sources, collection periods, test delivery methods, or study quality. Meta‐regression further clarified these findings, showing that differences in data sources and study quality accounted for 45% of the variability in the effects of rurality on participation. In comparison, 76% of the variability in the impact of remoteness was explained by factors such as data sources, the COVID‐19 period, and test delivery methods. The COVID‐19 pandemic emerged as a significant contextual factor, with disruptions to health services, reduced access to screening, and public restrictions, which may likely contribute to the widening of geographical disparities in participation [93].

### 4.1 Strengths

A key strength of this review is its international scope, covering studies from four regions and providing separate analyses based on varying definitions of geographical measures. This allows for comparing less dense and highly dense participation rates in Europe and rural versus urban areas across the Americas and Asia, as well as differences between remote and metropolitan areas in Australia.

Additionally, this review included subgroup analyses and meta‐regression. A meta‐regression analysis was also performed to further address the observed heterogeneity, based on data sources, study quality, publication year, the COVID‐19 period, and test delivery methods.

### 4.2 Limitations

Despite its strengths, this review has limitations. First, there was significant heterogeneity between studies, despite subgroup analysis and meta‐regression, due to the global scope and inconsistent definitions of “rural” and “urban” across regions and even within countries. This variability limits direct comparisons and weakens the generalisability of the pooled estimate. Additionally, the binary (rural/urban and remote vs. metropolitan) geographical classifications are unable to capture the actual differences, diversity, and complexity of rural environments, particularly in cross‐national comparisons. Second, although our review focuses solely on FIT‐based screening, the most recommended method, many studies lack clear screening methods used; although we attempted to contact the authors via email for clarification, only six responded (Table S2). Third, using raw data for consistency limited the ability to adjust for confounders, compromising the precision.

Third, varied definitions of rurality across regions prevented a pooled geographical estimate and led to overrepresentation from countries like Australia [28]. In contrast, countries with less geographical variation, such as the Netherlands, had fewer studies. Only two studies from the USA met the inclusion criteria due to mixed screening methods. Fourth, the absence of eligible studies from Africa restricts the generalisability of the findings to that region. This highlights a gap in the literature and shows the need for future research on geographical disparity in FIT‐based CRC screening in African populations. Finally, restricting to English‐language publications and the complexity of search terms may have excluded relevant articles.

## 5 Conclusions

This review highlights notable geographical disparities in FIT‐based CRC screening participation, particularly between rural and urban areas within countries. Specifically, higher participation in rural areas of Europe, with lower uptake in remote areas of Australia, reflects context‐specific barriers that influence screening behaviours. In contrast, no significant rural–urban differences were found in Asian countries. Additionally, there was no geographical disparity in FIT positivity rates between rural and urban areas.

These findings emphasise the importance of target, region‐specific interventions to promote equitable CRC screening. Future research should move beyond broad rural–urban comparisons to explore local area disparities, considering capturing differences within rural environments and the underlying factors contributing to them. The insights from this review can support policymakers and program managers in designing context‐specific strategies to address geographical variations in CRC prevention efforts.

## Author Contributions

Melkalem Mamuye Azanaw, Erin L. Symonds, Geraldine Laven‐Law, Wudneh Simegn Belay, Syme Aftab, and Molla M. Wassie contributed to the search process, including title and abstract screening, full‐text review, and study inclusion decisions. Melkalem Mamuye Azanaw, Erin L. Symonds, Geraldine Laven‐Law, Wudneh Simegn Belay, Syme Aftab, and Molla M. Wassie conducted risk‐of‐bias assessments and data extractions in pairs. Melkalem Mamuye Azanaw performed the meta‐analysis and drafted the manuscript, and Melkalem Mamuye Azanaw, Erin L. Symonds, Geraldine Laven‐Law, Wudneh Simegn Belay, Syme Aftab, Muktar B. Ahmed, and Molla M. Wassie critically reviewed and edited the original manuscript. Molla M. Wassie, Erin L. Symonds, and Muktar B. Ahmed supervised this work. All authors contributed to the manuscript and approved the final manuscript.

## Funding

The authors have nothing to report.

## Ethics Statement

The authors have nothing to report.

## Consent

The authors have nothing to report.

## Conflicts of Interest

The authors declare no conflicts of interest.

## Supporting information


**Table S1:** Search terms and strategy through six databases.
**Table S2:** List of excluded articles with reasons for exclusion (*n* = 306).
**Table S3:** Study quality assessment using modified Newcastle‐Ottawa quality appraisal elements for included articles (*n* = 35).
**Table S4:** Summary of variations in the overall participation rate for faecal immunochemical test‐based colorectal cancer screening by geographical regions (2005–2024).
**Table S5:** Summary of variations in participation rate for faecal immunochemical test‐based colorectal cancer screening across rural and urban areas in Europe, Asia, and the USA.
**Table S6:** Summary of variations in the participation rate for faecal immunochemical test‐based colorectal cancer screening by remoteness index in Australia.
**Table S7:** Subgroup analysis of the effects of rurality and remoteness on participation rates in faecal immunochemical test‐based colorectal cancer screening by different characteristics in geographical regions (2005–2024).
**Table S8:** Univariate Meta‐Regression of effects of rurality and remoteness on participation rates in faecal immunochemical test‐based colorectal cancer screening by different characteristics.
**Table S9:** Multivariable Meta‐Regression of effects of rurality and remoteness on participation rates in faecal immunochemical test‐based colorectal cancer screening by different characteristics.
**Table S10:** Summary of positivity rates in faecal immunochemical test‐based colorectal cancer screening by different characteristics.
**Table S11:** Positivity rates in faecal immunochemical test‐based colorectal cancer screening by rural–urban for the seven articles (2012–2024).
**Figure S1:** Descriptive comparison of participation rates based on population density level and rural–urban by considering a specific data collection period.
**Figure S2:** Descriptive comparison of participation rates based on remoteness index by considering a specific data collection period.
**Figure S3:** Counter‐enhanced funnel plot for effects of rurality on faecal immunochemical test‐based colorectal cancer screening participation rate in Europe, supported by a statistical test to assess publication bias.
**Figure S4:** Counter‐enhanced funnel plot for effects of rurality on faecal immunochemical test‐based colorectal cancer screening participation rate, supported by a statistical test to assess publication bias.
**Figure S5:** Forest plot of random effects meta‐analysis of effect estimates on faecal immunochemical test‐based colorectal cancer screening participation.
**Figure S6:** Counter‐enhanced funnel plot for effects of rurality on faecal immunochemical test‐based colorectal cancer screening positivity rate to assess publication bias.
**Figure S7:** Forest plot of random effects meta‐analysis of faecal immunochemical test‐based colorectal cancer screening positivity rate participation rate by different threshold levels.

## Data Availability

The data that support the findings of this study are available from the corresponding author upon reasonable request.
