# Supplementary material for: Geographical Disparities in Faecal Immunochemical Test‐Based Colorectal Cancer Screening Participation and Positivity Rates: A Systematic Review and Meta‐Analysis
Source: Health Promot J Austr. 2026 Mar 1;37(2):e70168. doi: 10.1002/hpja.70168 (PMC12950327; doi:10.1002/hpja.70168)
Supplement: Supplementary file 1 — Table S1: Search terms and strategy through six databases. Table S2: List of excluded articles with reasons for exclusion (n = 306). Table S3: Study quality assessment using modified Newcastle‐Ottawa quality appraisal elements for included articles (n = 35). Table S4: Summary of variations in the overall participation rate for faecal immunochemical test‐based colorectal cancer screening by geographical regions (2005–2024). Table S5: Summary of variations in participation rate for faecal immunochemical test‐based colorectal cancer screening across rural and urban areas in Europe, Asia, and the USA. Table S6: Summary of variations in the participation rate for faecal immunochemical test‐based colorectal cancer screening by remoteness index in Australia. Table S7: Subgroup analysis of the effects of rurality and remoteness on participation rates in faecal immunochemical test‐based colorectal cancer screening by different characteristics in geographical regions (2005–2024). Table S8: Univariate Meta‐Regression of effects of rurality and remoteness on participation rates in faecal immunochemical test‐based colorectal cancer screening by different characteristics. Table S9: Multivariable Meta‐Regression of effects of rurality and remoteness on participation rates in faecal immunochemical test‐based colorectal cancer screening by different characteristics. Table S10: Summary of positivity rates in faecal immunochemical test‐based colorectal cancer screening by different characteristics. Table S11: Positivity rates in faecal immunochemical test‐based colorectal cancer screening by rural–urban for the seven articles (2012–2024). Figure S1: Descriptive comparison of participation rates based on population density level and rural–urban by considering a specific data collection period. Figure S2: Descriptive comparison of participation rates based on remoteness index by considering a specific data collection period. Figure S3: Counter‐enhanced funnel plot for effects of ruralit [file HPJA-37-0-s001.docx]

**Supplementary Material**

**Geographical Disparities in Faecal Immunochemical Test-Based Colorectal Cancer Screening Participation and Positivity Rates: A Systematic Review and Meta-analysis**

**List of supplementary tables**

[Table S1: Search terms and strategy through six databases 2](#_Toc201427556)

[Table S2: List of excluded articles with reasons for exclusion (n=306) 8](#_Toc201427557)

[Table S3:Study quality assessment using modified Newcastle-Ottawa quality appraisal elements for included articles (n=35) 11](#_Toc201427558)

[Table S4: Summary of variations in the overall participation rate for faecal immunochemical test-based colorectal cancer screening by geographical regions (2005-2024) 13](#_Toc201427559)

[Table S5: Summary of variations in participation rate for faecal immunochemical test-based colorectal cancer screening across rural and urban areas in Europe, Asia, and the USA. 13](#_Toc201427560)

[Table S6: Summary of variations in the participation rate for faecal immunochemical test-based colorectal cancer screening by remoteness index in Australia 14](#_Toc201427561)

[Table S7: Subgroup analysis of effects of less dense, rurality and remoteness on participation rates in faecal immunochemical test-based colorectal cancer screening by different characteristics in geographical regions (2005-2024) 15](#_Toc201427562)

[Table S8: Univariate Meta-Regression of effects of rurality and remoteness on participation rates in faecal immunochemical test-based colorectal cancer screening by different characteristics. 17](#_Toc201427563)

[Table S9: Multivariable Meta-Regression of effects of rurality and remoteness on participation rates in faecal immunochemical test-based colorectal cancer screening by different characteristics 18](#_Toc201427564)

[Table S10: Summary of positivity rates in faecal immunochemical test-based colorectal cancer screening by different characteristics 19](#_Toc201427565)

[Table S11: Positivity rates in faecal immunochemical test-based colorectal cancer screening by rural-urban for the seven articles (2012-2024) 20](#_Toc201427566)

**List of supplementary figures**

[Figure S1: Descriptive comparison of participation rates based on population density level and rural-urban by considering specific data collection period 21](#_Toc201427571)

[Figure S2: Descriptive comparison of participation rates based on remoteness index by considering specific data collection period 22](#_Toc201427572)

[Figure S3: Counter-enhanced funnel plot for effects of population density on faecal immunochemical test-based colorectal cancer screening participation rate in Europe, supported by a statistical test to assess publication bias 23](#_Toc201427573)

[Figure S4: Counter-enhanced funnel plot for effects of rurality on faecal immunochemical test-based colorectal cancer screening participation rate, supported by statistical test to assess publication bias 24](#_Toc201427574)

[Figure S5: Forest plot of random effects meta-analysis of estimates effects on faecal immunochemical test-based colorectal cancer screening participation. 25](#_Toc201427575)

[Figure S6: Counter-enhanced funnel plot for effects of rurality on faecal immunochemical test-based colorectal cancer screening positivity rate to assess publication bias 26](#_Toc201427576)

[Figure S7: Forest plot of random effects meta-analysis of faecal immunochemical test-based colorectal cancer screening positivity rate participation rate by different threshold levels 27](#_Toc201427577)

Table S1: Search terms and strategy through six databases

| 1. MEDLINE(OVID) Database (Searched completed on 05/06/2024) | | | |  |
| --- | --- | --- | --- | --- |
| Concepts | SH and keyword | Original search (Medline) | Master search | Total searches |
| Colorectal cancer | 1 | Colorectal Neoplasms/ | Colorectal Neoplasms/ | 116331 |
|  | 2 | ((colorect* or rectal* or colon* or bowel*) adj3 (cancer* or neoplas* or carcino* or tumo?r* or adenocarcinoma* or adenom* or lesion* or malignant*)).tw,kf. | ((colorect* OR rectal* OR colon* OR bowel*) adj3 (cancer* OR neoplas* OR carcino* OR tumo?r* OR adenocarcinoma* OR adenom* OR lesion* OR malignant*)) | 296378 |
|  | 3 | 1 or 2 | 1 OR 2 | 310861 |
| Faecal immunochemical test | 4 | exp Occult Blood/ | exp Occult Blood/ | 6493 |
|  | 5 | Mass Screening/mt, sn [Methods, Statistics & Numerical Data] | Mass Screening/mt, sn [Methods, Statistics & Numerical Data] | 44157 |
|  | 6 | Feces/an, bl, ch, di [Analysis, Blood, Chemistry, Diagnosis] | Feces/an, bl, ch, di [Analysis, Blood, Chemistry, Diagnosis] | 27428 |
|  | 7 | Early Detection of Cancer/ | Early Detection of Cancer/ | 40614 |
|  | 8 | ((immunochem* or immuno-chem* or immunohistochem* or immuno-histochem* or Immunol* or immunochromatographic or immuno-chromatographic or immunoassay or immuno assay) adj4 (f?ecal or f?eces or stool or stools)).tw,kf. | ((immunochem* OR immuno-chem* OR immunohistochem* OR immuno-histochem* OR Immunol* OR immunochromatographic OR immuno-chromatographic OR immunoassay OR immuno assay) adj4 (f?ecal OR f?eces OR stool OR stools)) | 3152 |
|  | 9 | (iFOBT or FOBT or f?ecal occult blood test*).tw,kf. | (iFOBT OR FOBT OR f?ecal occult blood test*) | 4213 |
|  | 10 | ("FIT positive predictive value" or "FIT threshold*" or "fit positivity rate*" or "FIT positive*" or "FIT negative*" or "CRC participation rate").tw,kf | ("FIT positive predictive value" OR "FIT threshold*" OR "fit positivity rate*" OR "FIT positive*" OR "FIT negative*" OR "CRC participation rate") | 300 |
|  | 11 | ((Colorectal or Colon or rectal or bowel or large intestin* or lower intestin*) adj3 screen*).tw,kf. | ((Colorectal OR Colon OR rectal OR bowel OR large intestin* OR lower intestin*) adj3 screen*) | 14187 |
|  | 12 | 4 or 5 or 6 or 7 or 8 or 9 or 10 or 11 | 4 OR 5 OR 6 OR 7 OR 8 OR 9 OR 10 OR 11 | 117429 |
| Geographical disparity | 13 | Geography/ | Geography/ | 44037 |
|  | 14 | Geography, Medical/ | Geography, Medical/ | 1372 |
|  | 15 | Health Services Accessibility/ | Health Services Accessibility/ | 87764 |
|  | 16 | Health Status Disparities/ | Health Status Disparities/ | 20335 |
|  | 17 | Health Services/ | Health Services/ | 28203 |
|  | 18 | socioeconomic disparities in health/ | socioeconomic disparities in health/ | 238 |
|  | 19 | Spatial Analysis/ | Spatial Analysis/ | 5918 |
|  | 20 | Spatio-Temporal Analysis/ | Spatio-Temporal Analysis/ | 6423 |
|  | 21 | Spatial Regression/ | Spatial Regression/ | 354 |
|  | 22 | Geographic Information Systems/ | Geographic Information Systems/ | 9640 |
|  | 23 | Geographic Mapping/ | Geographic Mapping/ | 1626 |
|  | 24 | Rural Health/ | Rural Health/ | 23982 |
|  | 25 | Rural Population/ | Rural Population/ | 70775 |
|  | 26 | Rural Health Services/ | Rural Health Services/ | 14255 |
|  | 27 | Residence Characteristics/sn [Statistics & Numerical Data] | Residence Characteristics/sn [Statistics & Numerical Data] | 6804 |
|  | 28 | Urban population/ | urban population/ | 63315 |
|  | 29 | Urban Health/ | Urban Health/ | 18367 |
|  | 30 | Urban Health Services/ | Urban Health Services/ | 3796 |
|  | 31 | population density/ | population density/ | 24642 |
|  | 32 | Cities/ | Cities/ | 31426 |
|  | 33 | ((health status or health-status or healthcare or health-care or socioeconomic or socio-economic) adj3 disparit*).tw,kf. | ((health status OR health-status OR healthcare OR health-care OR socioeconomic OR socio-economic) adj3 disparit*) | 12199 |
|  | 34 | ((geograph* or spatial or spatiotemporal or spatio-temporal) adj3 analy*).tw,kf. | ((geograph* OR spatial OR spatiotemporal OR spatio-temporal) adj3 analy*) | 31200 |
|  | 35 | (urban-rural or rural* or urban* or remot* or suburban* or outback or cit* or metropolitan or less dens* or urban dens*).tw,kf. | (urban-rural OR rural* OR urban* OR remot* OR suburban* OR outback OR cit* OR metropolitan OR less dens* OR urban dens*) | 903113 |
|  | 36 | 13 or 14 or 15 or 16 or 17 or 18 or 19 or 20 or 21 or 22 or 23 or 24 or 25 or 26 or 27 or 28 or 29 or 30 or 31 or 32 or 33 or 34 or 35 | 13 OR 14 OR 15 OR 16 OR 17 OR 18 OR 19 OR 20 OR 21 OR 22 OR 23 OR 24 OR 25 OR 26 OR 27 OR 28 OR 29 OR 30 OR 31 OR 32 OR 33 OR 34 OR 35 | 1155407 |
| Combined | 37 | 3 and 12 and 36 | 3 AND 12 AND 36 | 1851 |

1. **CINAHL (EBSCOhost) (search completed on 06/06/2024)**

| Concepts | SH and keyword | Master search | CINAHL | Total searches |
| --- | --- | --- | --- | --- |
| Colorectal cancer | 1 | Colorectal Neoplasms/ | (MH "Colorectal Neoplasms") | 30,915 |
|  | 2 | ((colorect* OR rectal* OR colon* OR bowel*) adj3 (cancer* OR neoplas* OR carcino* OR tumo?r* OR adenocarcinoma* OR adenom* OR lesion* OR malignant*)) | TI ( ((colorect* OR rectal* OR colon* OR bowel* ) N3 (cancer* OR neoplas* OR carcino* OR tumo#r* OR adenocarcinoma* OR adenom* OR lesion* OR malignant* )) ) AND AB ( ((colorect* OR rectal* OR colon* OR bowel* ) N3 (cancer* OR neoplas* OR carcino* OR tumo#r* OR adenocarcinoma* OR adenom* OR lesion* OR malignant* )) ) | 22,424 |
|  | 3 | 1 OR 2 | S1 OR S2 | 39,291 |
| Faecal immunochemical test | 4 | Occult Blood/ | (MH "Occult Blood") | 1906 |
|  | 5 | Mass Screening/mt, sn [Methods, Statistics & Numerical Data] | (MH "Cancer Screening") | 18232 |
|  | 6 | Feces/an, bl, ch, di [Analysis, Blood, Chemistry, Diagnosis] | (MH "Feces/AN") | 3207 |
|  | 7 | Early Detection of Cancer/ | (MH "Early Detection of Cancer") | 13101 |
|  | 8 | ((immunochem* OR immuno-chem* OR immunohistochem* OR "immuno-histochem*" OR Immunol* OR immunochromatographic OR "immuno-chromatographic" OR immunoassay OR "immuno assay") adj4 (f?ecal OR f?eces OR stool OR stools)) | TI ( ((immunochem* OR immuno-chem* OR immunohistochem* OR immuno-histochem* OR Immunol* OR immunochromatographic OR immuno-chromatographic OR immunoassay OR "immuno assay" ) N4 (f#ecal OR f#eces OR stool OR stools )) ) OR AB ( ((immunochem* OR immuno-chem* OR immunohistochem* OR immuno-histochem* OR Immunol* OR immunochromatographic OR immuno-chromatographic OR immunoassay OR "immuno assay" ) N4 (f#ecal OR f#eces OR stool OR stools )) ) | 964 |
|  | 9 | (iFOBT OR FOBT OR "f?ecal occult blood test*") | TI ( (iFOBT OR FOBT OR "f#ecal occult blood test*" ) ) OR AB ( (iFOBT OR FOBT OR "f#ecal occult blood test*" ) ) | 1317 |
|  | 10 | ("FIT positive predictive value" OR "FIT threshold*" OR "fit positivity rate*" OR "FIT positive*" OR "FIT negative*" OR "CRC participation rate") | TI ( ("FIT positive predictive value" OR "FIT threshold*" OR "fit positivity rate*" OR "FIT positive*" OR "FIT negative*" OR "CRC participation rate" ) ) OR AB ( ("FIT positive predictive value" OR "FIT threshold*" OR "fit positivity rate*" OR "FIT positive*" OR "FIT negative*" OR "CRC participation rate" ) ) | 94 |
|  | 11 | ((Colorectal OR Colon OR rectal OR bowel OR "large intestin*" OR "lower intestin*") adj3 screen*) | TI ( ((Colorectal OR Colon OR rectal OR bowel OR "large intestin*" OR "lower intestin*" ) N3 screen* ) ) OR AB ( ((Colorectal OR Colon OR rectal OR bowel OR "large intestin*" OR "lower intestin*" ) N3 screen* ) ) | 6217 |
|  | 12 | 4 OR 5 OR 6 OR 7 OR 8 OR 9 OR 10 OR 11 | S4 OR S5 OR S6 OR S7 OR S8 OR S9 OR S10 OR S11 | 36,211 |
| Geographical disparity | 13 | Geography/ | (MH "Geographic Locations") | 8518 |
|  | 14 | Geography, Medical/ | (MH "Geographic Factors") | 20684 |
|  | 15 | Health Services Accessibility/ | (MH "Health Services Accessibility") | 111510 |
|  | 16 | Health Status Disparities/ | (MH "Health Status Disparities") | 10267 |
|  | 17 | Health Services/ | (MH "Health Services") | 16007 |
|  | 18 | socioeconomic disparities in health/ | (MH "Socioeconomic Disparities in Health") | 287 |
|  |  | Spatial Analysis/ |  |  |
|  |  | Spatio-Temporal Analysis/ |  |  |
|  |  | Spatial Regression/ |  |  |
|  | 19 | Geographic Information Systems/ | (MH "Geographic Information Systems") | 3647 |
|  | 20 | Geographic Mapping/ | (MH "Maps") | 3518 |
|  | 21 | Rural Health/ | (MH "Rural Health") | 7146 |
|  | 22 | Rural Population/ | (MH "Rural Population") | 13166 |
|  | 23 | Rural Health Services/ | (MH "Rural Areas") | 27545 |
|  | 24 | Residence Characteristics/sn [Statistics & Numerical Data] | (MH "Residence Characteristics") | 15406 |
|  | 25 | urban population/ | (MH "Urban Population") | 63315 |
|  | 26 | Urban Health/ | (MH "Urban Health") | 3958 |
|  | 27 | Urban Health Services/ | (MH "Urban Areas") | 25909 |
|  | 28 | population density/ | (MH "Population Density") | 925 |
|  |  | Cities/ |  |  |
|  | 30 | (("health status" OR "health-status" OR healthcare OR "health-care" OR socioeconomic OR "socio-economic") adj3 disparit*) | TI ( ("health status" OR health-status OR healthcare OR health-care OR socioeconomic OR socio-economic ) N3 disparit* ) ) OR AB ( ("health status" OR health-status OR healthcare OR health-care OR socioeconomic OR socio-economic ) N3 disparit* ) ) | 5397 |
|  | 31 | ((geograph* OR spatial OR spatiotemporal OR "spatio-temporal") adj3 analy*) | TI ( ((geograph* OR spatial OR spatiotemporal OR spatio-temporal ) N3 analy* ) ) OR AB ( ((geograph* OR spatial OR spatiotemporal OR spatio-temporal ) N3 analy* ) ) | 4213 |
|  | 32 | ("urban-rural" OR rural* OR urban* OR remot* OR suburban* OR outback OR cit* OR metropolitan OR "less dens*" OR "urban dens*") | TI ( (urban-rural OR rural* OR urban* OR remot* OR suburban* OR outback OR cit* OR metropolitan OR "less dens*" OR "urban dens*" ) ) OR AB ( (urban-rural OR rural* OR urban* OR remot* OR suburban* OR outback OR cit* OR metropolitan OR "less dens*" OR "urban dens*" ) ) | 258054 |
|  | 33 | 13 OR 14 OR 15 OR 16 OR 17 OR 18 OR 19 OR 20 OR 21 OR 22 OR 23 OR 24 OR 25 OR 26 OR 27 OR 28 OR 29 OR 30 OR 31 OR 32 OR 33 OR 34 OR 35 | S13 OR S14 OR S15 OR S16 OR S17 OR S18 OR S19 OR S20 OR S21 OR S22 OR S23 OR S24 OR S25 OR S26 OR S27 OR S28 OR S29 OR S30 OR S31 OR S32 | 430,266 |
| Combined | 37 | 3 AND 12 AND 36 | S3 AND S12 AND S33 | 899 |

1. **Scopus (Search completed on 06/06/2024)**

| Concepts | Search strings | # of articles |
| --- | --- | --- |
| Colorectal cancer | TITLE-ABS-KEY ((( colorect* OR rectal* OR colon* OR bowel* ) W/3 ( cancer* OR neoplas* OR carcino* OR tumour* OR adenocarcinoma* OR adenom* OR lesion* OR malignant* ) ) ) | 460,921 |
| Faecal immunochemical test | ((immunochem* OR immuno-chem* OR immunohistochem* OR immuno-histochem* OR Immunol* OR immunochromatographic OR immuno-chromatographic OR immunoassay OR "immuno assay" ) W/4 (faecal OR faeces OR stool OR stools )) OR (iFOBT OR FOBT OR "fecal occult blood test*" ) OR ("FIT positive predictive value" OR "FIT threshold*" OR "fit positivity rate*" OR "FIT positive*" OR "FIT negative*" OR "CRC participation rate" ) OR (((Colorectal OR Colon OR rectal OR bowel OR "large intestin*" OR "lower intestin*" ) W/3 screen* )) | 21089 |
| Geographical disparity | (("health status" OR health-status OR healthcare OR health-care OR socioeconomic OR socio-economic) W/3 disparit* ) OR ((geograph* OR spatial OR spatiotemporal OR spatio-temporal ) W/3 analy* ) OR (urban-rural OR rural* OR urban* OR remot* OR suburban* OR outback OR cit* OR metropolitan OR "less dens*" OR "urban dens*") | 3992138 |
| Total | S1 AND S2 AND S3 | 1786 |
| 1. Scopus query link (Search completed on 06/06/2024) =1789 | |  |
| TITLE-ABS-KEY ( ( ( colorect* OR rectal* OR colon* OR bowel* ) W/3 ( cancer* OR neoplas* OR carcino* OR tumour* OR adenocarcinoma* OR adenom* OR lesion* OR malignant* ) ) ) AND TITLE-ABS-KEY ( ( ( immunochem* OR immuno-chem* OR immunohistochem* OR immuno-histochem* OR immunol* OR immunochromatographic OR immuno-chromatographic OR immunoassay OR "immuno assay" ) W/4 ( faecal OR faeces OR stool OR stools ) ) OR ( ifobt OR fobt OR "fecal occult blood test*" ) OR ( "FIT positive predictive value" OR "FIT threshold*" OR "fit positivity rate*" OR "FIT positive*" OR "FIT negative*" OR "CRC participation rate" ) OR ( ( ( colorectal OR colon OR rectal OR bowel OR "large intestin*" OR "lower intestin*" ) W/3 screen* ) ) ) AND TITLE-ABS-KEY ( ( ( "health status" OR health-status OR healthcare OR health-care OR socioeconomic OR socio-economic ) W/3 disparit* ) OR ( ( geograph* OR spatial OR spatiotemporal OR spatio-temporal ) W/3 analy* ) OR ( urban rural OR rural* OR urban* OR remot* OR suburban* OR outback OR cit* OR metropolitan OR "less dens*" OR "urban dens*" ) ) ) | | |
| 1. Web of Science query link ((Search completed on 24/06/2024) =1299 | | |
| **((colorect* OR rectal* OR colon* OR bowel* ) NEAR/3 (cancer* OR neoplas* OR carcino* OR tumo$r* OR adenocarcinoma* OR adenom* OR lesion* OR malignant* ))** (Topic) and **((immunochem* OR immuno-chem* OR immunohistochem* OR immuno-histochem* OR Immunol* OR immunochromatographic OR immuno-chromatographic OR immunoassay OR "immuno assay" ) NEAR/4 (f$ecal OR f$eces OR stool OR stools )) OR (iFOBT OR FOBT OR "f$ecal occult blood test*" ) OR ("FIT positive predictive value" OR "FIT threshold*" OR "fit positivity rate*" OR "FIT positive*" OR "FIT negative*" OR "CRC participation rate" ) OR ((Colorectal OR Colon OR rectal OR bowel OR "large intestin*" OR "lower intestin*" ) NEAR/3 screen* )** (Topic) and **(("health status" OR health-status OR healthcare OR health-care OR socioeconomic OR socio-economic ) NEAR/3 disparit* ) OR ((geograph* OR spatial OR spatiotemporal OR spatio-temporal ) NEAR/3 analy* ) OR (urban-rural OR rural* OR urban* OR remot* OR suburban* OR outback OR cit* OR metropolitan OR "less dens*" OR "urban dens*" )** (Topic) | | |
| 1. ****ProQuest query search string ((Search completed on 22/06/2024)** =**749**** | | |
| noft((colorect* OR rectal* OR colon* OR bowel*) NEAR/3 (cancer* OR neoplas* OR carcino* OR tumo*r* OR adenocarcinoma* OR adenom* OR lesion* OR malignant*)) AND noft(((immunochem* OR immuno-chem* OR immunohistochem* OR immuno-histochem* OR Immunol* OR immunochromatographic OR immuno-chromatographic OR immunoassay OR "immuno assay") NEAR/4 (f*ecal OR f?eces OR stool OR stools)) OR (iFOBT OR FOBT OR "f*ecal occult blood test*") OR ("FIT positive predictive value" OR "FIT threshold*" OR "fit positivity rate*" OR "FIT positive*" OR "FIT negative*" OR "CRC participation rate") OR ((Colorectal OR Colon OR rectal OR bowel OR "large intestin*" OR "lower intestin*") NEAR/3 screen*)) AND noft((("health status" OR health-status OR healthcare OR socioeconomic OR socio-economic) NEAR/3 disparit*) OR ((geograph* OR spatial OR spatiotemporal OR spatio-temporal) NEAR/3 analy*) OR (urban-rural OR rural* OR urban* OR remot* OR suburban* OR outback OR cit* OR metropolitan OR "less dens*" OR "urban dens*")) AND la.exact("ENG") | | |

Table S2: List of excluded articles with reasons for exclusion (n=306)

| Study reference | Specific reason | Reason for Exclusion | Excluded | Total excluded | Percent |
| --- | --- | --- | --- | --- | --- |
| #447 - Abdel-Rahman 2021, #7139 - Alatise 2022, #1165 - Mansouri 2013, #171 - Levitz 2023, #1197 - Thorpe 2013, #68 - Adefemi 2024, #255 - GiorgiRossi 2023, #876 - Ko 2016, #763 - Domingo 2018, #252 - Centra 2023, #1018 - Crouse 2015, #178 - Kives 2022, #283 - Bright 2022, #435 - Zhan 2021, #346 - Vives 2022, #1211 - Wan 2015, #8633 - Hurtado 2015, #1634 - Schootman 2006, #7424 Schreuders, #8968 - Sherman 2012, #701 - Dacus 2018, #2618 - Llanos 2015, #1416 - Pandhi 2010, #1086 - Sherman 2014, #5347 - Elder 2017, #558 - Dominic 2020, #7534 - Durkin 2019, 752 - Preston 2018, #1708 - Cole 2003, #4292 - Haverkamp 2020, #309 - Ritzenthaler 2022, #815 - Best 2017, #387 - Bhimla 2021, #615 - Blair 2019, #5577 - Christy 2016, #7281 - Cardoso 2020, #472 - Coronado 2021, #5976 - Deutekom 2009, #1715 - Cibula 2003, #1215 - Lawson 2015, #7234 - Deeds 202, #6747 - Clark 2020, #807 - Deding 2017, #7172 - Selby 2022, #3241 - Martellucci 2022, #5211 - Maxwell 2016, #7254 - Doubeni 2016, #1331 - Fleming 2011, #8787 - Hillyer 2014, #1452 - Felix 2009, #580 - Gonzalez 2020, #1739 - Powe 2002, #7447 - Tepeš 2017, 1618 - Anonymous 2006, #446 - Nielsen 2021, #245 - BarberanParraga 2023, #982 - Manne 2015, 846 - Caron 2018, #536 - Koivogui 2020, ##5600 - Gorini 2023, #116 - Moss 2024, #222 - Worthington 2023, #20 - Yeary 2023, #560 - Chow 2020, #1469 - Zittleman 2009, #506 - Conn 2020  #2219 - Triantafillidis 2010, #106 - Peng 2024, #6229 - Sandiford 2019, #1200 - Lin 2013, #6749 - Symonds 2015, #2669 - Coronado 2009, #374 - SyedSoffian 2021, #1740 - Janda 2002, #492 - Smith 2020, #520 - Hirko 2020, #585 - Hong 2020, #1700 - Greiner 2004, #740 - Woodall 2018, #272 - Davis 2023, #852 - Davis 2017, #79 - Katz 2024, #1604 - Basch 2006, #340 - Beaney 2022, #469 - Elangovan 2021, #1183 - Liss 2013, #6900 - Hughes 2016, #144 - Ribeiro 2023 #683 - Buehler 2019, #159 - Bongaerts 2023, #177 - Guo 2023 | No results by comparing urban and rural geographical areas (n=90) | Studies only in one geographical area (only urban or rural) or geography is not mentioned (93) | No results by geography | 135 | 44.3 |
| #2892 - Girardi 2024, #7151 - Moss 2017, #261 - McClellan 2023 | Only in one geographical region |  |  |  |  |
| #412 - Bandi 2021, #7214 - Pellat 2021, #949 - Calo 2015, #7112 - Campbell 2020, #7943 - Cataneo 2022, #3937 - Digby 2012, #134 - Creavin 2023, #365 - Earl 2022, #470 - Gawron 2021, #669 - WarrenAndersen 2019, #5654 - Nguyen 2015, #5690 - Selva 2019 #446 - Nielson 2021, #1350 - Wee 2011, #1271 - Wee 2012, #136, #828 - Siantz 2017, #5584 - Stoffel 2022, #1178 - Tan 2013, #301 - Rose 2022, #6803 - Zorzi 2020, #1422 - Singh 2010, #452 - Genovese 2021, #4688 - Jerant 2008, #3526 - Rebolj 2020, #1081 - Pruitt 2014, #3527 - Rao 2016, #1054 - Pignone 2014, #7308 - Parente 2011, #1090 - Wang 2014, #842 - Kelly 2019, #5403 - Melvin 2019, #8790 - Solmi 2015, #7222 - Stegeman 2015, #856 - Mema 2017, #1426 - Weller 2010, #1422 - Singh 2010, #2669 - Coronado 2009, #5600 - Gorini 2023, #8790 - Solmi 2015, #3241 - Martellucci 2022, #134 - Creavin 2023, #387 - Bhimla 2021, #7424-Schreuders, #1263 - Joseph 2012, #5211 - Maxwell 2016 | Geography is not mentioned. | Geography is not mentioned (42) |  |  |  |
| #993 - Hughes 2015, #55 - Aruma 2024, #1179 - Lofters 2013, #686 - Lofters 2019, #9328 - Ko 2005, #413 - Geddes 2021 | Author responded | Not FIT-based screening after the author's response | Not FIT-based screening | 6 | 1.9 |
| #392 - Abdus 2021, #497 - Alyabsi 2020, #642 - Blair 2019, #161 - Borders 2023, #1421 - Brennenstuhl 2010, #806 - Davis 2017, #1257 - Davis 2012, #1292 - Bennett 2012, #208 - Daniel 2021, #9271 - Fisher 2007, #377 - Guo 2021 | No separate data for the FIT results | Excluded after author's nonresponse | CRC screening with unspecified methods | 40 | 13.1 |
| #39 - Ashad-Bishop 2023, #375 - Weisband 2021, #50 - Calatayud 2024, #126 - BaekerBispo 2024, #51 - Mobley 2012, #2694 - Hoff 2008, #80 - Honaker 2024, #7604 - Ryu 2014, #314 - Sabatino 2022, #1065 - Horner-Johnson 2014, #1288 - Myong 2012, #572 - Mojica 2020, , #1154 - Hui 2013, #1628 - James 2006, #298 - Theodoropoulos 2022, #695 - Liaw 2018, #157 - Huang 2023, #7117 - Lin 2017, #357 - Moss 2022, #511 - Lee 2020, #7047 - Kong 2022, #4245 - VahabiMandana 2021, #315 - Park 2022, , #4312 - Marcinek 2020, #295 - Bauer 2022 | Results by FOBT |  |  |  |  |
| #504 - Salinas 2020, #2709 - Gonzales 2012, #1476 - Hao 2009 | FIT and colonoscopy |  |  |  |  |
| #207 - Zhu 2021, #1539 - Schumacher 2008, #857 - Wang 2017, #151 - Smayda 2023, #1360 - Trivedi 2011, #6829 - Pornet 2010, #1349 - Whitaker 2011, #1716 - Gondal 2003, #1070 - Towne 2014, #2268 - Swiatkowski 2012, #1005 - Berkowitz 2015, #1737 - Bretthauer 2002, #1529 - Shih 2008, #1609 - Rutledge 2006, #142 - Gunn 2023 | Sigmoidoscopy/colonoscopy | Due to the nature of the data source | Not FIT-based screening | 67 | 22.0 |
| #6030 - Wardle 2016, #1783 - Tazi 1997, #1184 - FonSing 2013, #937 - Manca 2015, #9024 - Moss 2012, #9073 - vonWagner 2011, #1826 - Eliakim 1988, #1105 - Lo 2014, #3832 - Lo 2013, #6835 - Hirst 2018, #5346 - Guillaume 2017, #1799 - Herbert 1995 | gFOBT-based screening |  |  |  |  |
| #964 - Faruque 2015, #1542 - Weissman 2008, #17 - Tsai 2024, #1718 - Nelson 2003, #1313 - Bennett 2011, #516 - Moss 2020, #53 - Liang 2008, #1009 - Ojinnaka 2015, #583 - Carmichael 2020, #386 - Shete 2021, #3400 - Chen 2021, #1235 - Cole 2012, #1163 - Cole 2013, #874 - Feng 2016, #1699 - Coughlin 2004, #707 - Hendryx 2018, #303 - Hirko 2022, #2486 - McDaniel 2019 | BRFSS data- unable to find separate data for screening methods |  |  |  |  |
| #1807 - Launoy 1993, #2214 - MANT 1992, #1392 - LeRetraite 2010 | Hemoccult test or colonoscopy |  |  |  |  |
| #658 - Simkin 2019, #1186 - Modiri 2013, #676 - Moss 2019, #1790 - Herbert 1997, #1507 - Lian 2008, #1362 - Gobl 2011, #49 - Goetz 2024, #1251 - Kreuter 2012, #4109 - Jessiman-Perreault 2023, #855 - Al-Thafar 2017, 1080 - Aboagye 2014, #7047 - Kong 2022, #1694 - Goldberg 2004, #1411 - Nnoaham 2010, #80 - Honaker 2024, #982 - Manne 2015, #609 - Gavens 2019 | Survey data- unable to find separate data for screening methods |  |  |  |  |
| #235 - Brenner 2023 | Preferences for pharmacy | Wrong study design/outcome |  | 20 | 6.6 |
| #1309 - Daley 2012 | Qualitative study |  |  |  |  |
| #1203 - Shahidi 2013, #8312 - Sanders 2020, #9506 - Morris 2012, #245 - BarberanParraga 2023, #553 - Holle 2020, #1094 - Schlichting 2014, #621 - Czaderny 2019, #6756 - Högberg 2022, #3598 - Rich 2011, #1745 - Hawley 2001, #792 - LePimpec 2017, #131 - Gupta 2023, #1143 - Davis 2013, #1086 - Sherman 2014 | Wrong outcome |  |  |  |  |
| #46 - Horshauge 2020, #384 - Cubiella 2021 | Program based |  |  |  |  |
| #546 - Chen 2020, # Davis 2013, #629 - Jen 2019 | Symptomatic patients |  |  |  |  |
| #1798 - Mahon 1995, #1426 - Weller 2010, #4175 - WWu 2022, #796 - Olver 2017, | Editorial comments | Not correct publication | Wrong study characteristics | 37 | 12.1 |
| #4170 - AhmedAbdulelah 2022 | Poster presentation |  |  |  |  |
| #1797 - Perez-Stable 1995, #2283 - Charlton 2010 | Conference Paper |  |  |  |  |
| #1013 - Fraser 2015, #797 - Mostafazadeh-Bora 2017, #4767 - Nelson 2002, #8157-anonyms | Letter to the Editor |  |  |  |  |
| #4607 - Honein 2012, #7249 - Jalili 2024, #2433 - McLeod 2022, #4395 - Mehta 2018, #2252 - Lian 2008, #2291 - Swaminathan 2020, #2328 - James 2017, #1861 - Arnold 2018, #2373 - Waclawik 2023, #2288 - Theodoropoulos 2022, #2320 - Wolbert 2019, #4359 - Rodriguez Berrios 2019, #718 - Juul 2018, #7478 - Carethers 2015, #178 - Kives 2022, #54 - Ma 2024, #2418 - Dominic 2010, #133 - Castaneda 2023, #324 - Barsouk 2022, #283 - Bright 2022, | Thesis or Abstract |  |  |  |  |
| #1217 - Young 2015 | Study protocol |  |  |  |  |
| #2646 - Launoy 1996, #1793 - Launoy 1996 | Article in French | Not in the English language |  |  |  |
| #8553 - Yang 2017, #8085 - Cao 2021 | Article in Chinese |  |  |  |  |

BRFSS= Behavioral Risk Factor Surveillance System

Table S3:Study quality assessment using modified Newcastle-Ottawa quality appraisal elements for included articles (n=35)

| **Study reference** | Country | **Selection** | | | | | | **Comparability** | **Outcome** | | | | **Score** | **Quality** |
| --- | --- | --- | --- | --- | --- | --- | --- | --- | --- | --- | --- | --- | --- | --- |
|  |  | R | S | AE | O | NS | SS | CC | OA | LF | AF | Statistics |  |  |
| Baum (2023) | Australia | 1 | NA | 2 | NA | 1 | 1 | 1 | 2 | NA | NA | 0 | 8 | Good |
| Bright (2023) | Wales | 1 | NA | 2 | NA | 1 | 1 | 2 | 2 | NA | NA | 1 | 10 | Excellent |
| Dasgupta (2023) | Australia | 1 | NA | 2 | NA | 1 | 1 | 2 | 2 | NA | NA | 0 | 9 | Excellent |
| DeKlerk (2022) | Netherlands | 1 | NA | 2 | NA | 1 | 1 | 0 | 2 | NA | NA | 1 | 8 | Good |
| Fletcher (2024) | Australia | 1 | NA | 1 | NA | 1 | 1 | 2 | 2 | NA | NA | 1 | 9 | Excellent |
| Fukuda (2005) | Japan | 0 | NA | 2 | NA | 1 | 1 | 2 | 1 | NA | NA | 1 | 8 | Good |
| Giorgi Rossi (2018) | Italy | 1 | NA | 1 | NA | 1 | 1 | 2 | 1 | NA | NA | 1 | 7 | Good |
| Gong (2018) | China | 1 | NA | 2 | NA | 1 | 1 | 0 | 2 | NA | NA | 0 | 7 | Good |
| Goodwin (2019) | Australia | 1 | NA | 1 | NA | 1 | 1 | 1 | 1 | NA | NA | 1 | 7 | Good |
| Goodwin (2020) | Australia | 1 | NA | 2 | NA | 1 | 1 | 2 | 2 | NA | NA | 1 | 9 | Excellent |
| Irwin (2024) | Australia | 1 | NA | 2 | NA | 1 | 1 | 1 | 2 | NA | NA | 0 | 8 | Good |
| Lin (2019) | China | 1 | NA | 2 | NA | 1 | 1 | 1 | 1 | NA | NA | 0 | 7 | Good |
| LoConte 2013 | USA | 0 | NA | 2 | NA | 1 | 0 | 0 | 2 | NA | NA | 0 | 5 | Satisfactory |
| Martini (2011) | Australia | 1 | NA | 2 | NA | 1 | 1 | 2 | 1 | NA | NA | 0 | 8 | Good |
| Nikbakht (2018) | Iran | 1 | NA | 2 | NA | 1 | 1 | 2 | 1 | NA | NA | 1 | 9 | Excellent |
| Slimings (2021) | Australia | 1 | NA | 2 | NA | 1 | 1 | 2 | 2 | NA | NA | 1 | 9 | Excellent |
| Salimzadeh (2017) | Iran | 1 | NA | 2 | NA | 1 | 0 | 1 | 2 | NA | NA | 0 | 7 | Good |
| Stracci (2019) | Italy | 1 | NA | 2 | NA | 0 | 1 | 2 | 2 | NA | NA | 0 | 6 | Satisfactory |
| Sun (2018) | Australia | 1 | NA | 2 | NA | 1 | 1 | 2 | 2 | NA | NA | 1 | 10 | Excellent |
| Trinh (2022) | Korea | 1 | NA | 1 | NA | 1 | 1 | 1 | 1 | NA | NA | 0 | 6 | Satisfactory |
| Dimova (2015) | Bulgaria | 1 | NA | 2 | NA | 1 | 1 | 2 | 0 | NA | NA | 0 | 7 | Good |
| VanHal (2011) | Belgium | 1 | NA | 1 | NA | 1 | 1 | 1 | 2 | NA | NA | 0 | 6 | Satisfactory |
| Roosbroeck (2012) | Belgium | 1 | NA | 2 | NA | 1 | 0 | 2 | 2 | NA | NA | 1 | 8 | Good |
| Varlow (2014) | Australia | 1 | NA | 1 | NA | 1 | 1 | 1 | 2 | NA | NA | 0 | 7 | Good |
| Ward (2011) | Australia | 1 | NA | 2 | NA | 0 | 1 | 2 | 2 | NA | NA | 1 | 9 | Excellent |
| Cebrino (2023) | Spain | 1 | NA | 2 | NA | 1 | 1 | 2 | 2 | NA | NA | 1 | 10 | Excellent |
| Dancourt (2016) | France | 1 | 1 | 1 | 1 | 1 | NA | 2 | 1 | 1 | 1 | NA | 9 | Excellent |
| Hol (2010) | Netherlands | 1 | 1 | 1 | 1 | 1 | NA | 2 | 1 | 0 | 0 | NA | 7 | Good |
| Kregting (2022) | Netherlands | 1 | 1 | 1 | 1 | 1 | NA | 1 | 1 | 1 | 0 | NA | 7 | Good |
| Manuc (2024) | Romania | 1 | 1 | 1 | 1 | 1 | NA | 1 | 1 | 1 | 1 | NA | 8 | Good |
| O'Connor (2020) | USA | 1 | 1 | 1 | 1 | 1 | NA | 1 | 1 | 0 | 1 | NA | 7 | Good |
| Pornet (2014) | France | 1 | 1 | 2 | 1 | 1 | NA | 1 | 1 | 1 | 1 | NA | 9 | Excellent |
| Schliemann (2023) | Malaysia | 1 | 1 | 2 | 1 | 1 | NA | 2 | 1 | 1 | 0 | NA | 9 | Excellent |
| Ramai (2019) | USA | 0 | 1 | 2 | 1 | 1 | NA | 1 | 1 | 1 | 1 | NA | 8 | Good |

Key:

- R = Representativeness; S= Selection of the non-exposed cohort; AE=Ascertainment of exposure; O=outcome of interest at the start of the study; NS = Non-respondents; Sample size justified; CC = Confounding controlled; OA = Outcome assessment; LF=follow-up long enough; AF=Adequacy of follow-up of cohorts; Statistics
- For survey/cross-sectional study: R, AE, NS, SS, CC, OA and statistics
- For follow-up/cohort study: R, S, AE, O, CC, OA, LF and AF
- Scores
- Very Good Studies: 9-10 points
- Good Studies: 7-8 points
- Satisfactory Studies: 5-6 points
- Unsatisfactory Studies: 0 to 4 points

Table S4: Summary of variations in the overall participation rate for faecal immunochemical test-based colorectal cancer screening by geographical regions (2005-2024)

| Geographical regions | Number of included articles | Number of Invited Participants | Number of screened participants | Participation (95% CI) | I^2^ (%) |
| --- | --- | --- | --- | --- | --- |
| Overall | 33 | 22,000,413 | 9,397,611 | 49.9 (40.6, 59.2) | 100 |
| Australia | 11 | 16,324,541 | 6,809,372 | 45.2 (39.6, 50.9) | 100 |
| Europe | 12 | 3,333,876 | 2,225,675 | 61.9 (49.3, 73.1) | 100 |
| Asia | 6 | 2,309,329 | 356,746 | 36.4 (7.3, 80.6) | 99.7 |
| America | 3 | 32,667 | 5,818 | 47.0 (1.8, 97.7) | 99.9 |

** From the nine included articles, a total of 11 data collection periods were included to calculate participation rates*

Table 5: Summary of variations in participation rate for faecal immunochemical test-based colorectal cancer screening across rural and urban areas in Europe, Asia, and the USA.

|  |  | | Geographical classification | | | | |
| --- | --- | --- | --- | --- | --- | --- | --- |
| Author Last name (Year) | Total | | Rural area | | Urban area | | Odds ratio (95%CI) |
|  | Invited (n) | Screened, n (%) | Invited (n) | Screened, n (%) | Invited (n) | Screened, n (%) |  |
| Europe | | | | | | | |
| Bright (2023) | 55,459 | 33,497 (60.4) | 33,644 | 20,624 (61.3) | 18,723 | 11253 (60.1) | 1.05 (1.01,1.09) |
| Kregting (2022) | 332,484 | 249,535 (75.1) | 72,940 | 57,802 (79.2) | 145,936 | 112,876 (77.3) | 1.12 (1.09,1.14) |
| Cebrino (2023) 1 | 7,234 | 2,340 (42.1) | 3671 | 1226 (33.4) | 2445 | 1,114 (31.3) | 1.10 (1.00,1.22) |
| Cebrino (2023) 2 | 6,929 | 3,043(43.9) | 4062 | 1,824 (44.9) | 3,571 | 1,114 (42.3) | 1.11 (1.01,1.22) |
| Manuc (2024) | 168,958 | 152,083 (90.0) | 78346 | 71,953 (91.8) | 90612 | 80119 (88.4) | 1.47 (1.42,1.52) |
| Pornet (2014) | 118,905 | 78,975 (66.4) | 25461 | 17,500 (68.7) | 93444 | 61475 (65.8) | 1.14 (1.11.1.78) |
| Dimova (2015) | 600 | 473 (78.8) | 260 | 223 (85.8) | 340 | 250 (73.5) | 2.17 (1.42,3.13) |
| Asia | | | | | | | |
| Lin (2019) | 2,283,214 | 350,581 (15.4) | 782,287 | 124,614 (15.9) | 1500927 | 225,967 (15.1) | 1.07 (1.06,1.08) |
| Schliemann (2023) | 747 | 311(41.6) | 199 | 78 (39.2) | 548 | 233 (42.5) | 0.87 (0.63,1.21) |
| Y. Fukuda (2005) | 15,224 | 3,288(21.6) | 13156 | 2961 (22.5) | 2068 | 329 (15.9) | 1.53 (1.36,1.74) |
| Trinh (2022) | 4,500 | 860(19.1) | 441 | 66 (15.0) | 2057 | 399 (19.4) | 0.73 (0.55,0.97) |
| Trinh (2022) * | 4,600 | 704(15.3) | 382 | 40 (10.5) | 20384 | 375 (18.4) | 0.52 (0.37,0.74) |
| USA | | | | | | | |
| LoConte (2013) | 633 | 506(79.9) | 82 | 66 (80.0) | 551 | 440 (79.9) | 1.04 (0.58,1.86) |
| O’Connor (2020) | 30,667 | 4630(15.1) | 10,330 | 1,384 (13.4) | 19437 | 3,246 (16.7) | 0.77 (0.72,0.83) |

NOTE: Values in bold indicate P < 0.05

Table S6: Summary of variations in the participation rate for faecal immunochemical test-based colorectal cancer screening by remoteness index in Australia

| Author (Year) | Australian remoteness area classification | | | | | | | |
| --- | --- | --- | --- | --- | --- | --- | --- | --- |
|  | **Remote** | | **Rural*** | | **Inner regional** | | **Metropolitan** | |
|  | Invite (n) | Screen (%) | Invite (n) | Screen (n) | Invite (n) | Screen (n) | Invite (n) | Screen (n) |
| Dasgupta R1 (2023) | 44568 | 15599(35) | 345354 | 141486 | 650459 | 289105 | 2164677 | 863,851 |
| Dasgupta R2 (2023) | 77495 | 26475(34) | 563573 | 232506 | 1046458 | 476202 | 3449056 | 1,436,070 |
| Dasgupta R3 (2023) | 81624 | 27437(34) | 607693 | 252441 | 1172420 | 547146 | 3971707 | 1,722,590 |
| Fletcher (2024) |  |  | 1200 | 641 | 1871 | 1031 | 6385 | 3,588 |
| Goodwin (2019) |  |  | 52 | 30 | 107 | 61 | 201 | 128 |
| Goodwin (2020) |  |  | 914 | 309 | 1191 | 401 | 3483 | 1,104 |
| Irwin R1 (2024) | 2320 | 819(35) | 19554 | 8316 | 39047 | 18079 | 107902 | 46,290 |
| Irwin R2 (2024) | 2071 | 628(30) | 17882 | 6873 | 37299 | 16188 | 106377 | 43,189 |
| Martini (2011) | 2237 | 1029 (46) | 12096 | 5878 |  |  | 60449 | 27,573 |
| Sun (2018) | 25604 | 6958 (27.2) | 190897 | 62662 | 364791 | 133149 | 1299512 | 434,037 |
| Varlow (2014) |  |  | 563 | 354 |  |  | 1375 | 799 |
| Total | 235919 | 78944 | 1771874 | 717366 | 3313643 | 1481362 | 1771874 | 717366 |

*Key: ARIA+ classification: metropolitan (0-0.2), Inner regional (0.2-2.4), Rural (2.4-15), remote (5.92-15.53), *= rounds of data collection period*

*(R1=Round one, R2=Round two, R3=Round three), values in bold indicate P < 0.05*

Table S7: Subgroup analysis of the effects of rurality and remoteness on participation rates in faecal immunochemical test-based colorectal cancer screening by different characteristics in geographical regions (2005-2024)

| Study groups | Category | Europe | | | | Asia | | | |
| --- | --- | --- | --- | --- | --- | --- | --- | --- | --- |
|  |  | Articles (n) | OR (95%CI) | I^2^ (%) | P-value | Articles (n) | OR (95%CI) | I^2^ (%) | P-value |
| Primary included articles | | 7 | 1.20 (1.01,1.42) | 97.60 | 0.04 | 5 | 0.92 (0.71,1.20) | 93.00 | 0.55 |
| Data collection period | 2005-2015 | 2 | 1.52 (0.81,2.85) | 88.59 | 0.03 | 2 | 1.27 (0.89,1.74) | 96.80 | 0.18 |
|  | 2016-2024 | 5 | 1.17 (1.01,1.34) | 98.3 | 0.03 | 3 | 0.69 (0.53,0.92) | 56.60 | 0.01 |
|  | Subgroup differences | |  |  | 0.42 |  |  |  | 0.01 |
| COVID-19 period | Yes | 3 | 1.20 (0.76,1.89) | 98.9 | 0.01 | 1 | 0.52 (0.71, 0.73) | -- | 0.01 |
|  | No | 4 | 1.14 (0.99,1.30) | 71.98 | 0.01 | 4 | 1.04 (0.81,1.35) | 92.50 | 0.731 |
|  | Subgroup differences | |  |  | 0.62 |  |  |  | 0.01 |
| Study design | Cross-sectional | 4 | 1.13 (1.02, 1.25) | 76.24 | 0.12 | 4 | 0.93 (0.69,1.26) | 94.60 | 0.65 |
|  | Follow-up study/cohort | 3 | 1.23 (1.05,1.45) | 99.7 | 0.01 | 1 | 0.87 (0.63,1.21) | -- | 0.42 |
|  | Subgroup differences | |  |  | 0.33 |  |  |  | 0.77 |
| Study quality | Satisfactory |  |  |  |  | 2 | 0.63 (0.45,0.87) | 55.80 | 0.01 |
|  | Good | 3 | 1.42 (0.68,2.93) | 98.99 | 0.01 | 2 | 1.27 (0.89,1.81) | 96.90 | 0.181 |
|  | Excellent | 4 | 1.10 (1.04,1.16) | 75.20 | 0.01 | 1 | 0.87 (0.63,1.21) | --- | 0.42 |
|  | Subgroup differences | |  |  | 0.12 |  |  |  | 0.02 |

Australia

| Study groups |  | Remote vs metropolitan | | | | Rural vs metropolitan | | | | Inner regional vs. metropolitan | | | |
| --- | --- | --- | --- | --- | --- | --- | --- | --- | --- | --- | --- | --- | --- |
|  | Category | Articles (n) | OR (95%CI) | I^2^  (%) | P | Articles | OR (95%CI | I^2^ (%) | P | Articles | OR (95%CI | I2 (%) | P |
| Data collection period | 2006-2014 | 1 | 1.02 (0.93,1.11) | ----- | 0.719 | 4 | 1.08 (0.97,1.02) | 94.6 | 0.17 | 2 | 1.15 (1.13,1.15) | 0.0 | 0.01 |
|  | 2015-2024 | 6 | 0.72 (0.67,0.77) | 98.2 | 0.001 | 7 | 0.96 (0.92,1.01) | 99.1 | 0.12 | 7 | 1.15 (1.12,1.18) | 97.6 | 0.01 |
|  | Subgroup difference | |  |  | 0.001 |  |  |  | 0.06 |  |  |  | 0.85 |
| Study setting | National | 6 | 0.72 (0.67,0.77) | 98.2 | 0.001 | 8 | 0.98 (0.94,1.02) | 98.9 | 0.25 | 8 | 1.15 (1.13,1.18) | 97.3 | 0.01 |
|  | Reginal | 1 | 0.92 (0.71,1.20) | ---- | 0.719 | 3 | 1.06 (0.90,1.26) | 84.7 | 0.48 | 1 | 0.96 (0.86,1.06) | ---- | 0.40 |
|  | Subgroup difference | |  | -- | 0.001 |  |  |  | 0.33 |  |  |  | 0.01 |
| Data sources | Research survey | |  | ---- |  | 3 | 0.99 (0.77,1.29) | 71.5 | 0.96 | 2 | 0.95 (0.86,1.05) | 0.0 | 0.29 |
|  | Administrative | 7 | 0.75 (0.70,0.81) | 98.2 | 0.001 | 8 | 1.00 (0.96,1.03) | 99.0 | 0.81 | 7 | 1.16 (1.13,1.18) | 97.6 | 0.01 |
|  | Subgroup difference | |  |  | --- |  |  |  | 0.99 |  |  |  | 0.01 |
| COVID-19 period | Yes | 4 | 0.71 (0.64,0.98) | 98.9 | 0.03 | 5 | 0.96 (0.91,1.01) | 99.4 | 0.13 | 5 | 1.15 (1.12,1.18) | 98.1 | 0.01 |
|  | No | 3 | 0.82 (0.64,0.78) | 95.8 | 0.01 | 6 | 1.04 (0.98,1.12) | 91.1 | 0.21 | 4 | 1.15 (1.13,1.16) | 10.1 | 0.01 |
|  | Subgroup difference | |  | --- | 0.18 |  |  |  | 0.06 |  |  |  | 0.79 |
| Test delivery | Test mailed | 7 | 0.75 (0.70,0.81) | 98.2 | 0.01 | 8 | 0.98 (0.95,1.02) | 99.0 | 0.43 | 7 | 1.15 (1.13,1.17) | 97.7 | 0.01 |
|  | Self-report |  |  | ----- |  | 3 | 1.13 (1.01,1.27) | 0.98 | 0.05 | 2 | 0.98 (0.70,1.36) | 52.4 | 0.90 |
|  | Subgroup difference | |  | ----- | -- |  |  |  | 0.04 |  |  |  | 0.34 |
| Study quality | Good | 3 | 0.73 (0.67,0.80) | 96.3 | 0.01 | 5 | 0.98 (0.94,1.03) | 94.3 | 0.43 | 3 | 1.15 (1.13,1.18) | 55.9 | 0.01 |
|  | Excellent | 4 | 0.78 (0.59,1.02) | 98.9 | 0.07 | 6 | 1.02 (0.92,1.14) | 99.2 | 0.65 | 6 | 1.13 (1.10,1.17) | 98.1 | 0.01 |
|  | Subgroup difference | |  | 3.41 | 0.69 |  |  |  | 0.46 |  |  |  | 0.34 |

Table S8: Univariate Meta-Regression of effects of rurality and remoteness on participation rates in faecal immunochemical test-based colorectal cancer screening by different characteristics.

| Fixed effects | | Rurality in Europe | | | | Remoteness in Australia | | | |
| --- | --- | --- | --- | --- | --- | --- | --- | --- | --- |
| Variable | Category | Coefficient | (95%CI) | SE | P | Coefficient | (95%CI) | SE | P |
| Publication year | | -0.01 | (-0.05, 0.02) | 0.02 | 0.49 | -0.03 | (-0.04, -0.01) | 0.01 | 0.002* |
| Data sources | Survey | ref |  |  |  |  |  |  |  |
|  | Administrative | -0.62 | (-1.12, -0.13) | 0.25 | 0.01* | -0.21 | (-0.51,0.09) | 0.15 | 0.17* |
| COVID-19 period | No | ref |  |  |  |  |  |  |  |
|  | Yes | 0.01 | (- 0.29, 0.26) | 0.11 | 0.93 | -0.23 | (-0.43, -0.03) | 0.11 | 0.03* |
| Mail Test return | No | ref |  |  |  |  |  |  |  |
|  | Yes | 0.09 | (-0.24, 0.44) | 0.17 | 0.58 | -0.33 | (-0.59, -0.07) | 0.13 | 0.01* |
| Study quality | Good | ref |  |  |  |  |  |  |  |
|  | Excellent | -0.21 | (-0.41, -0.01) | 0.10 | 0.04* | -0.02 | (10.28,0.23) | 0.13 | 0.86 |

**Indicates inclusion in multiple meta-regression model (P-value<0.25); SE=standard error; P= P value*

Table S9: Multivariable Meta-Regression of effects of rurality and remoteness on participation rates in faecal immunochemical test-based colorectal cancer screening by different characteristics

| Fixed effects | | Rurality in Europe | | | | Remoteness in Australia | | | |
| --- | --- | --- | --- | --- | --- | --- | --- | --- | --- |
| Variable | Category | Coefficient | (95%CI) | SE | P | Coefficient | (95%CI) | SE | P |
| Constant | | 0.77 | (0.30,1.24) | 0.24 | 0.01* | 52.5 | (15.37,89.68) | 18.9 | 0.01 |
| Publication year | |  |  |  |  | -0.03 | (-0.04, -0.01) | 0.01 | 0.01 |
| Data sources | Survey | ref |  |  |  | ref |  |  |  |
|  | Administrative | -0.52 | (-1.01, -0.03) | 0.25 | 0.03* | -0.13 | (-0.34,0.07) | 0.10 | 0.19 |
| COVID-19 period | No |  |  |  |  | ref |  |  |  |
|  | Yes |  |  |  |  | -0.01 | (-0.18,0.21) | 0.10 | 0.90 |
| Mail Test return | No |  |  |  |  | ref |  |  |  |
|  | Yes |  |  |  |  | -0.24 | (-0.46, -0.01) | 0.11 | 0.04 |
| Study quality | Good | ref |  |  |  |  |  |  |  |
|  | Excellent | -0.15 | (-0.33, 0.03) | 0.09 | 0.10 |  |  |  |  |
| Random effects | | | | | | | | | |
| Residual heterogeneity (tau^2^) | | 0.01 |  |  | 0.02 | 0.009 |  |  | 0.001 |
| Unaccounted variability (I^2^) | | 96.34 |  |  |  | 97.7% |  |  |  |
| Sampling variability (H^2^) | | 27.35 |  |  |  | 44.7 |  |  |  |
| Accounted heterogeneity (R^2^) | | 48.85% |  |  |  | 76.2% |  |  |  |

Table S10: Summary of positivity rates in faecal immunochemical test-based colorectal cancer screening by different characteristics

| Study groups | Category | Included articles | Positivity rate (95% CI) | I^2^ (%) | P value |
| --- | --- | --- | --- | --- | --- |
| Overall screening positivity rates | | 14 | 8.7(6.5, 11.7) | 99.9 |  |
| Regional classification | Europe | 8 | 6.7 (4.9, 9.0) | 99.9 |  |
|  | America | 1 | 11.3 (8.8, 14.3) | --- |  |
|  | Asia | 5 | 11.3 (6.0, 23.8) | 99.9 |  |
|  | Subgroup differences | |  |  | 0.006 |
| Age range in years | 50-70 | 3 | 9.4 (7.4, 15.2) | 99.9 |  |
|  | 50-74 | 9 | 8.5 (5.1, 13.8) | 99.9 |  |
|  | 45-75 | 2 | 8.9 (5.8, 13.4) | 0.03 |  |
|  | Subgroup differences | |  |  | 0.892 |
| Organized program | Yes | 11 | 7.9 (5.6, 11.2) | 99.9 |  |
|  | No | 3 | 12.2 (4.8, 27.6) | 89.1 |  |
|  | Subgroup differences | |  |  | 0.096 |
| FIT brand | OC-Sensor | 7 | 7.7 (4.1, 13.8) | 98.5 |  |
|  | Sentinel diagnostics | 3 | 9.4 (5.6,15.2) | 99.0 |  |
|  | WPHM Hemosure | 2 | 8.3 (1.8, 8.1) | 100.0 |  |
|  | Subgroup differences | |  |  | 0.001 |
| Number of FIT samples | One | 5 | 7.8 (3.7,15.7) | 97.6 |  |
|  | Two | 3 | 12.3 (1.8, 50.8) | 100.0 |  |
|  | Subgroup differences | |  |  | 0.624 |
| Data sources | Administrative | 7 | 8.8 (5.5,13.5) | 99.9 |  |
|  | Research survey | 7 | 8.7 (5.0,14.7) | 98.8 |  |
|  | Subgroup differences | |  |  | 0.995 |
| Study quality | Good | 10 | 7.8 (6.1, 9.4) | 99.9 |  |
|  | Excellent | 3 | 12.8(10.4, 67.3) | 99.7 |  |
|  | Subgroup differences | |  |  | 0.036 |

Table S11: Positivity rates in faecal immunochemical test-based colorectal cancer screening by rural-urban for the seven articles (2012-2024)

| Author (Year) | Total | | Rural | | Urban | |  |
| --- | --- | --- | --- | --- | --- | --- | --- |
|  | Screened | Positive | Screened | Positive | Screened | Positive | OR (95%CI) |
| Bělobrádek (2024) | 392,257 | 29,027 | 99,986 | 7199 | 190,800 | 14,310 | **0.96 (0.93, 0.99)** |
| Bělobrádek (2024) | 320,623 | 33,986 | 83,358 | 8836 | 156,657 | 16,449 | 1.01 (0.98,1.04) |
| Bělobrádek (2024) | 365,019 | 37,962 | 97,165 | 10591 | 174,549 | 17,804 | **1.08 (1.05, 1.10)** |
| Dancourt (2016) | 20,371 | 865 | 11,946 | 526 | 8411 | 345 | 1.08 (0.94,1.24) |
| Gong (2018) | 809,528 | 88,222 | 267,098 | 41,248 | 542,430 | 46,974 | **1.93 (1.90.1.95)** |
| Lin (2019) | 350,581 | 21,808 | 124614 | 6647 | 225,967 | 15,161 | **0.78 (0.76,0.81)** |
| LoConte (2013) | 506 | 57 | 66 | 13 | 440 | 44 | **2.39 (1.25,4.57)** |
| Manuc (2024) | 152,083 | 8,334 | 71,953 | 3986 | 80,119 | 4,799 | 0.92 (0.88,0.96) |
| Nikbakht (2018) | 924 | 229 | 484 | 118 | 413 | 111 | 0.88 (0.65,1.18) |
| Total | 2,424,886 | 221,264 | 756,670 | 79,164 | 1,379,786 | 115,997 | 1.11 (0.83,1.47) |
| Pooled estimates | 9.4% (95% CI: 7.8, 11.1) | | 10.1%(95%CI:7.2,13.4) | | 8.5% (95% CI: 7.4, 9.7) | |  |

*Note: Values in bold indicate P < 0.05*

A

Figure S1: Descriptive comparison of participation rates based on population density level and rural-urban by considering a specific data collection period

Figure S2: Descriptive comparison of participation rates based on remoteness index by considering a specific data collection period


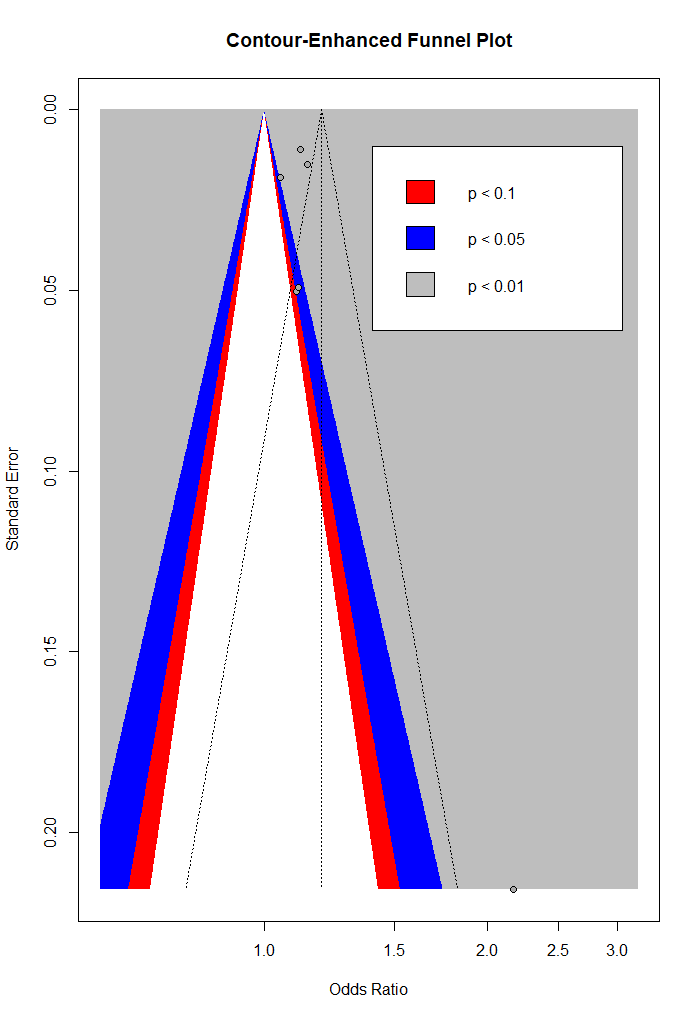


Figure S3: Counter-enhanced funnel plot for effects of rurality on faecal immunochemical test-based colorectal cancer screening participation rate in Europe, supported by a statistical test to assess publication bias

Meta bias (statistical test)

| Linear regression test of funnel plot asymmetry | | | |
| --- | --- | --- | --- |
| Bias estimate | SE | t | P value |
| 192.84 | 805.91 | 0.24 | 0.82 |


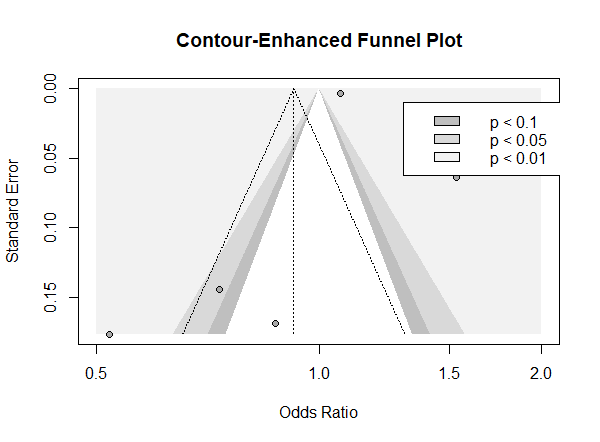


Meta bias (statistical test)

| Linear regression test of funnel plot asymmetry | | | |
| --- | --- | --- | --- |
| Bias estimate | SE | t | P value |
| -0.64 | 2.23 | -0.29 | 0.7918 |

Figure S4: Counter-enhanced funnel plot for effects of rurality on faecal immunochemical test-based colorectal cancer screening participation rate, supported by a statistical test to assess publication bias


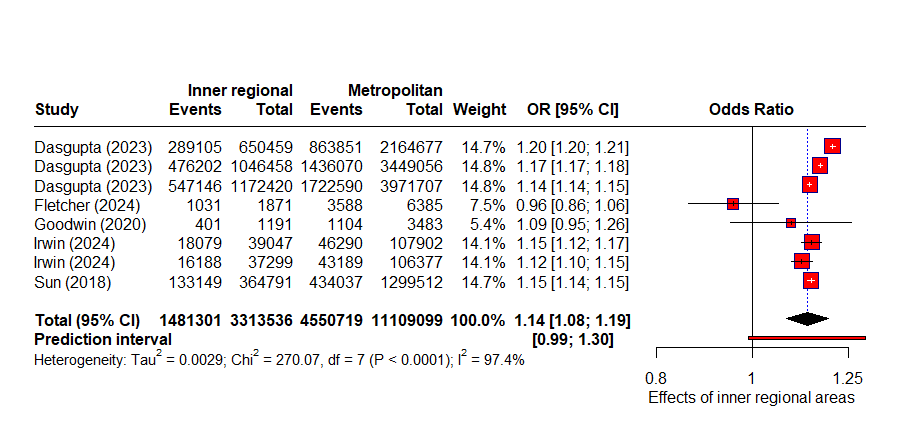


Figure S5: Forest plot of random effects meta-analysis of effect estimates on faecal immunochemical test-based colorectal cancer screening participation.


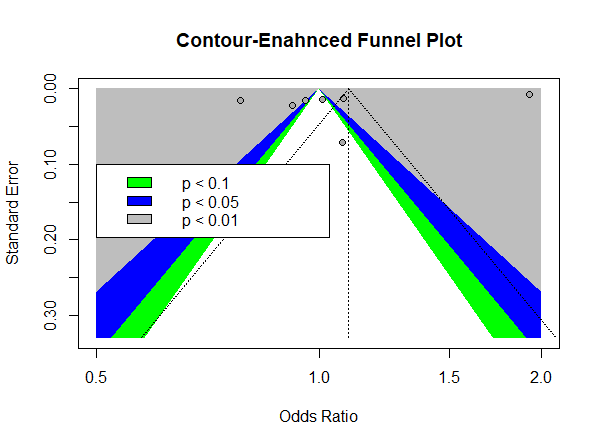


Figure S6: Counter-enhanced funnel plot for effects of rurality on faecal immunochemical test-based colorectal cancer screening positivity rate to assess publication bias

Figure S7: Forest plot of random effects meta-analysis of faecal immunochemical test-based colorectal cancer screening positivity rate participation rate by different threshold levels
